# Supplementary material for: N-terminal cysteine acetylation and oxidation patterns may define protein stability
Source: Nat Commun. 2024 Jun 25;15:5360. doi: 10.1038/s41467-024-49489-2 (PMC11199558; doi:10.1038/s41467-024-49489-2)
Supplement: Supplementary file 1 — Supplementary Information [file 41467_2024_49489_MOESM1_ESM.pdf]

N-terminal cysteine acetylation and oxidation patterns may define protein stability

Supplementary Figures and Tables

Karen C Heathcote[1,2,3], Thomas P Keeley[2], Matti Myllykoski[4], Malin Lundekvam[4], Nina McTiernan[4], Salma Akter[1], Norma Masson[2], Peter J Ratcliffe[2,3*], Thomas Arnesen[4,5*], Emily Flashman[6*]

1 – Department of Chemistry, University of Oxford, Oxford OX1 3TA, U.K.

2 – Ludwig Institute for Cancer Research, Nuffield Department of Medicine, University of Oxford, Oxford OX3 7FZ, U.K.

3 – The Francis Crick Institute, 1 Midland Road, London NW1 1AT, U.K.

4 – Department of Biomedicine, University of Bergen, Bergen 5020, Norway

5 – Department of Surgery, Haukeland University Hospital, Bergen 5021, Norway

6 – Department of Biology, University of Oxford, Oxford OX1 3RB, UK

*Corresponding authors

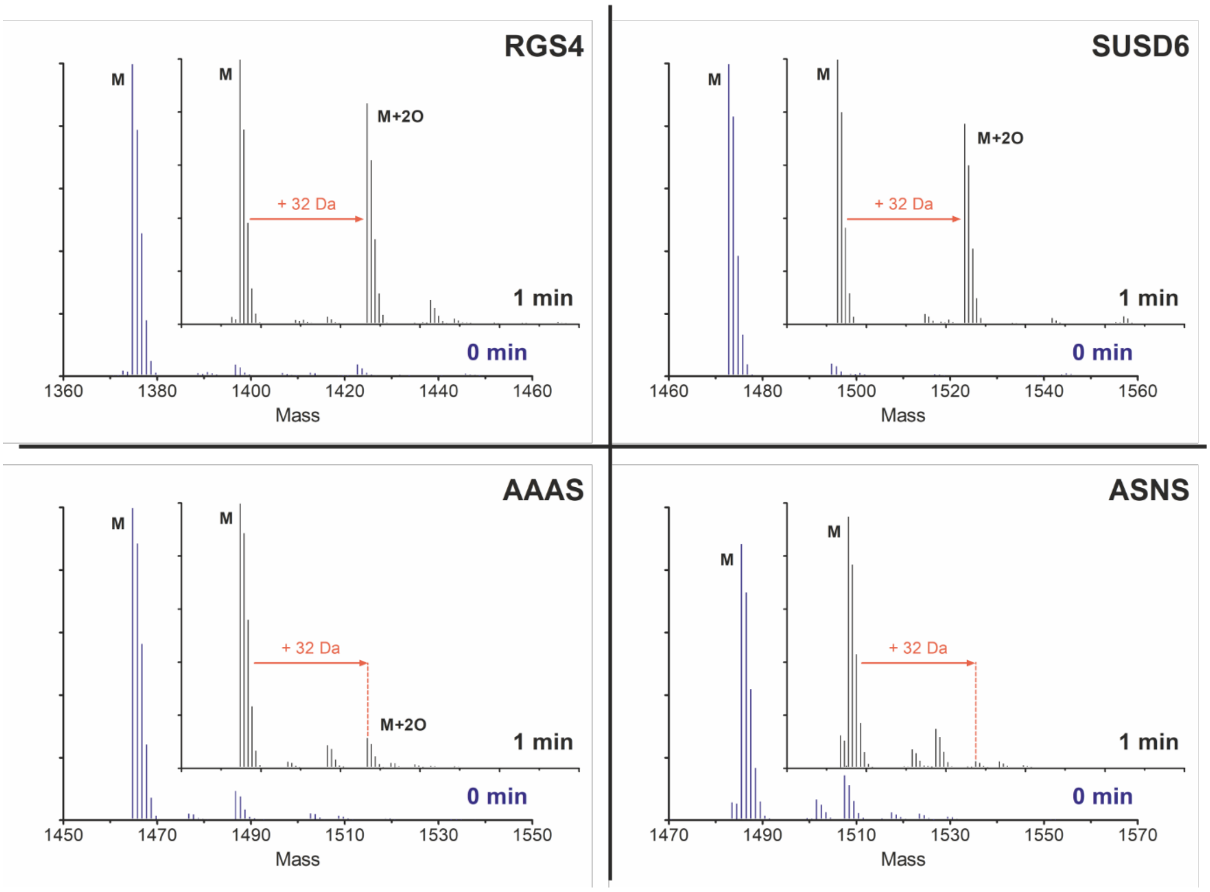

Supplementary Figure 1: Mass spectra of representative Nt-Cys peptides after incubation with ADO

Deconvoluted mass spectra showing oxidation (+32 Da shift) of $RGS4_{2\text{-}15}$ (M=1374.72), $SUSD6_{2\text{-}15}$ (M=1472.76), $AAAS_{2\text{-}15}$ (M=1464.76) and $ASNS_{2\text{-}15}$ (M=1485.50) peptides after 1-minute incubation with *Hs*ADO. Unmodified peptide (M) and oxidised peptide (M+2O) peaks indicated on spectra.

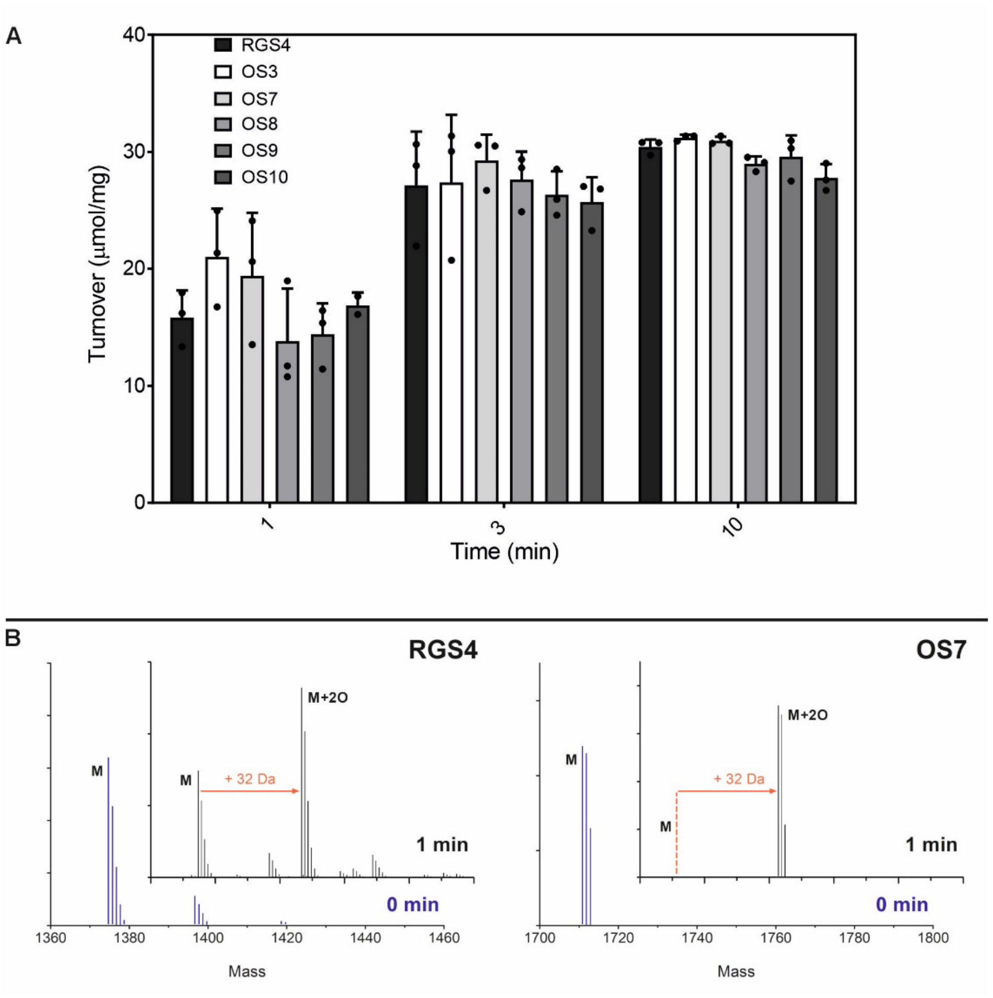

Supplementary Figure 2: Time-course showing oxidation of best OS peptides catalysed by ADO and mass spectra of oxidised OS7

A - Oxidation of best optimum substrate (OS) peptides in presence of ADO over 10 minutes. RGS4 included for comparison. Data represent mean +/- standard deviation, n=3.

B - Deconvoluted mass spectra (LCMS) of $RGS4_{2\text{-}15}$ and OS7 peptides after incubation with ADO. For OS peptide sequences, see Figure 2.

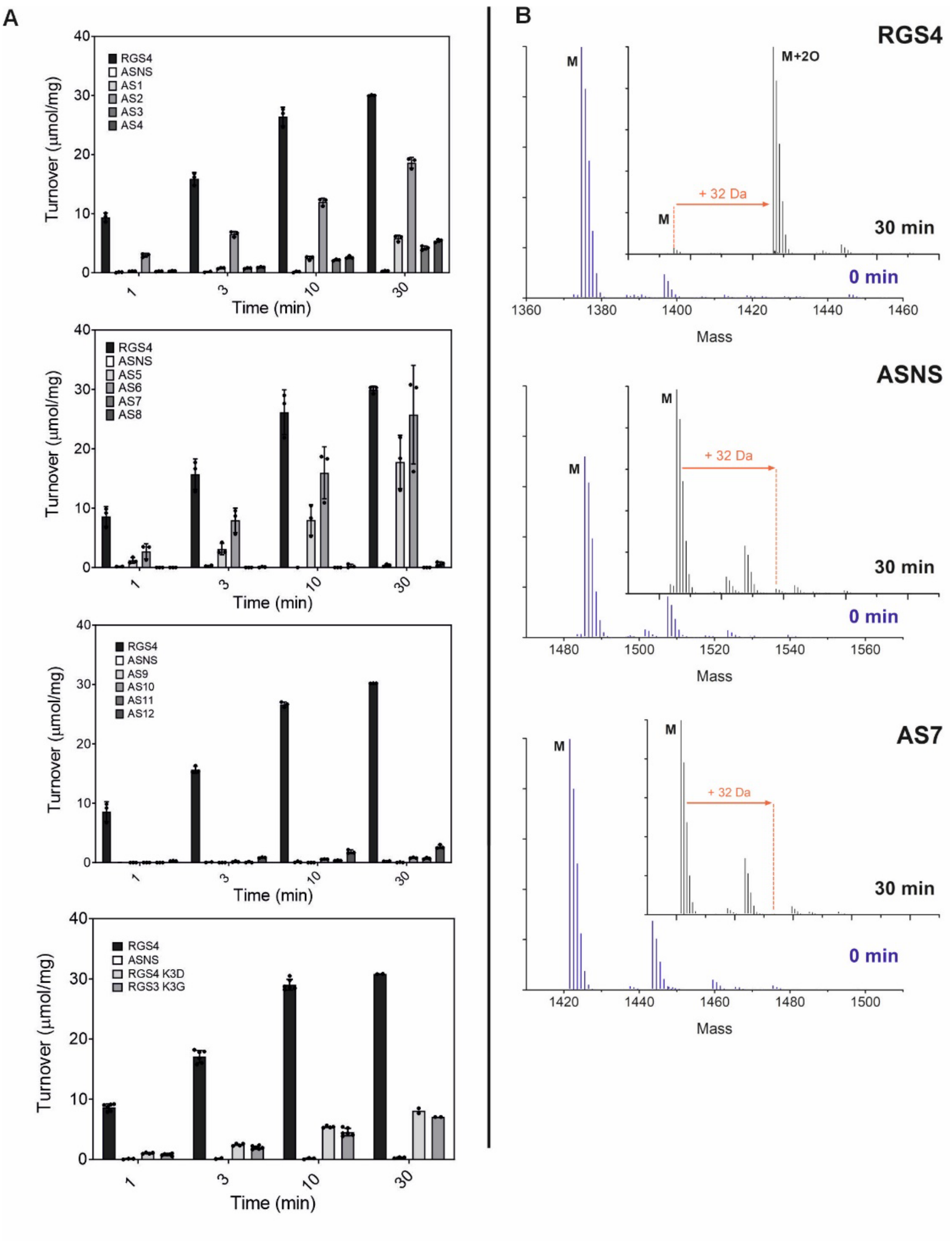

Supplementary Figure 3: Time-courses and mass spectra showing oxidation of AS peptides and RGS4 K3D/G catalysed by ADO

A - Oxidation of anti-substrate (AS) and RGS4 K3D/G peptides in the presence of ADO over 30 minutes. ASNS included as a negative control; RGS4 as a positive control. Data represent mean +/- standard deviation, n=3 except for RGS4 K3D (n=4; n=2@30 mins), RGS4 K3G (n=6; n=2@30 mins).

B - Deconvoluted mass spectra of $RGS4_{2-15}$, $ASNS_{2-15}$ and AS7 peptides after incubation with ADO. For AS peptide sequences, see Figure 3.

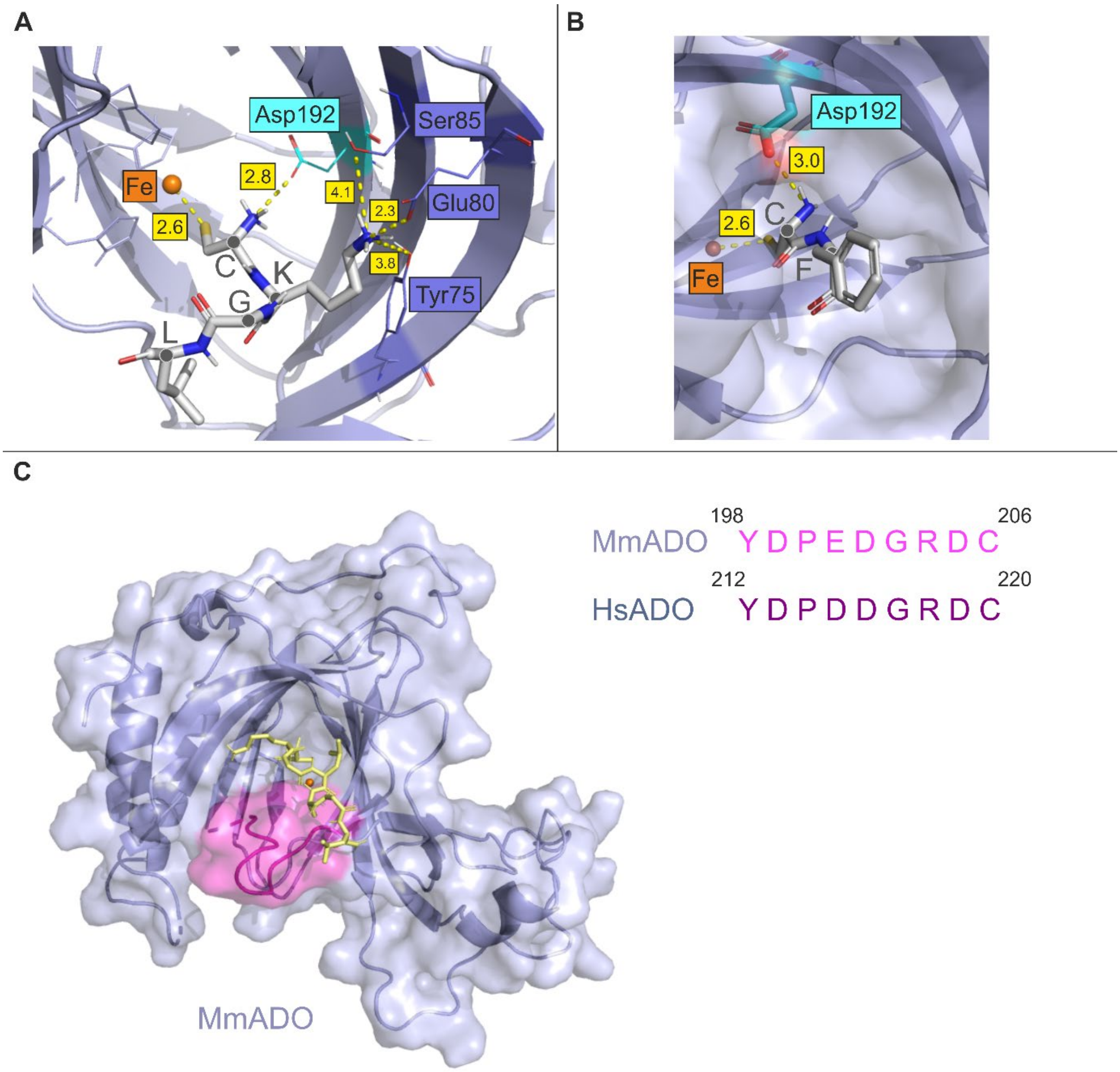

Supplementary Figure 4: ADO structure and AutoDock4 studies with substrate peptides

A - Docking result with RGS4$_{2-8}$ peptide (light grey sticks) showing residues 2-5 (CKGL) bound in active site of *Mm*ADO (PDB:7lvz chain D, light blue cartoon). Nt-Cys thiol bound to active site Fe (orange); Nt-amine bound to Asp192 (cyan lines); side chain amine of RGS4 Lys within a distance of 4.1 Å of polar pocket Ser85, Glu80, Tyr75 (slate blue). Potential bonding distances between RGS4$_{2-8}$ peptide atoms and *Mm*ADO side chain atoms are highlighted in yellow and given in Å units, α-Carbons marked with grey dots.

B - Docking result with IL32$_{2-8}$ peptide: residues 2-3 (CF) in active site of *Mm*ADO (PDB:7lvz chain D, light blue surface); showing a potential orientation of the Phe residue when Nt-amine is bound to Asp192 (cyan). Potential bonding distances between IL32$_{2-3}$ peptide atoms and *Mm*ADO side chain atoms are highlighted in yellow and given in Å units, α-Carbons marked with grey dots.

C - Docking result with $RGS4_{2\text{-}8}$ peptide bound to *Mm*ADO (7lvzD) showing peptide (CKGLAGL) (yellow sticks) and potential substrate binding (hairpin) loop (magenta). Sequences of hairpin loop in *Mm*ADO and *Hs*ADO in top right.

Supplementary Discussion: We considered whether there could be a link between ADO structure and the sequences of some of the least active peptide substrates. Peptides AS7-12 (with which ADO showed little to no activity under the conditions tested) all possess an 'EE' motif at residues 11 and 12. This motif of two acidic residues at position 11 is also observed in ASNS, which has 'DD' at this location. This is of particular interest as ASNS does not have acidic residues before position 11, suggesting this 'DD' motif could be responsible for ADO's low activity with this sequence. A potential substrate binding loop has previously been identified in ADO's structure, as well as in the PCOs (Fig. S4C) [1–4]. This loop sits near the entrance to the active site and is rich in acidic residues in ADO, but interestingly is not acidic in PCOs. In the docking experiments that we performed all ADO residues were set to be rigid; therefore, we did not consider this to be a useful experiment for exploring potential interactions of peptides with the hairpin loop, which we expect to be flexible. However, we think it is possible that residues in the C-terminal half of the 14 mer peptides could interact with the hairpin loop. Our docking experiment with $RGS4_{2\text{-}8}$ suggested that residues after position 7 would protrude from the main active site cavity and could be in close proximity to the hairpin loop (Fig S4C). This would help to explain ADO activity data with $ASNS_{2\text{-}15}$ and AS7-12 peptides, as a concentrated acidic region in the peptide C-terminus could repel the acidic residues in the hairpin loop and disfavour binding. It is worth noting that IL32 also possesses a 'DD' motif at position 9 in its 14-mer peptide; this is accompanied, however, by a 'KK' motif at position 12 and a particularly favourable N terminus (as noted above), which may explain why this peptide is still a good ADO substrate.

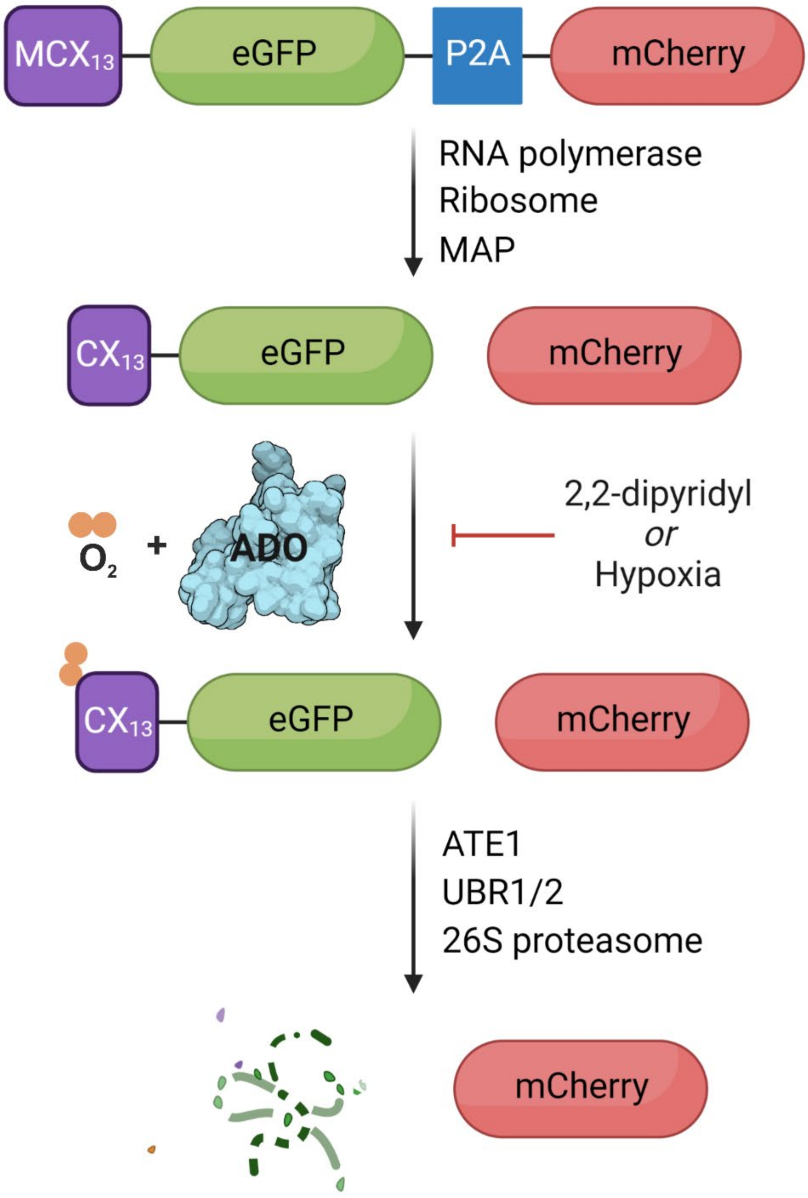

Supplementary Figure 5: Dual-Fluorescence Oxygen Reporter (DFOR) assay scheme[5]

MCXn-eGFP-mCherry plasmids are transfected into cells. eGFP and mCherry DNA sequences are separated by a self-cleaving P2A sequence; CXn-eGFP and mCherry protein levels should therefore be equivalent following protein translation and Met excision by methionine aminopeptidase (MAP). If the CXn N-terminal sequence is an ADO substrate then the Nt-Cys will be oxidised and the CXn-eGFP protein should be degraded via the Arg/N-degron pathway; eGFP fluorescence will be reduced relative to mCherry fluorescence so CXn-eGFP levels can be monitored by the ratio of eGFP/mCherry fluorescence. ATE1 = arginyl transferase; UBR1/2 = E3 ubiquitin-protein ligases.

Created using Biorender (license agreement JI26TL30S0).

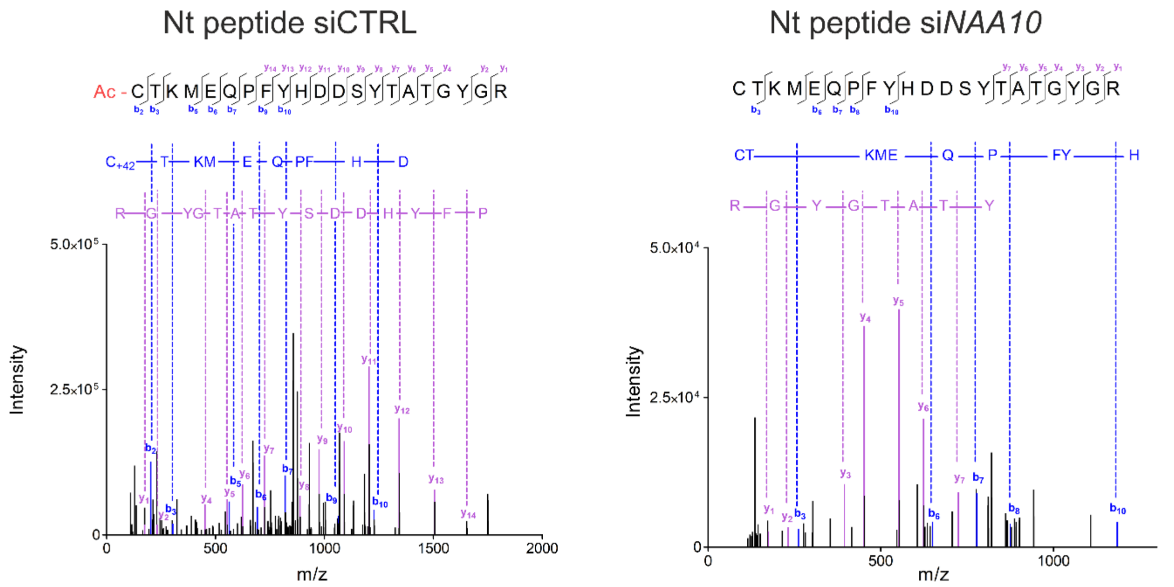

Supplementary Figure 6: Expanded view of JUNB N-terminal peptide MS/MS spectra (Fig. 5C)

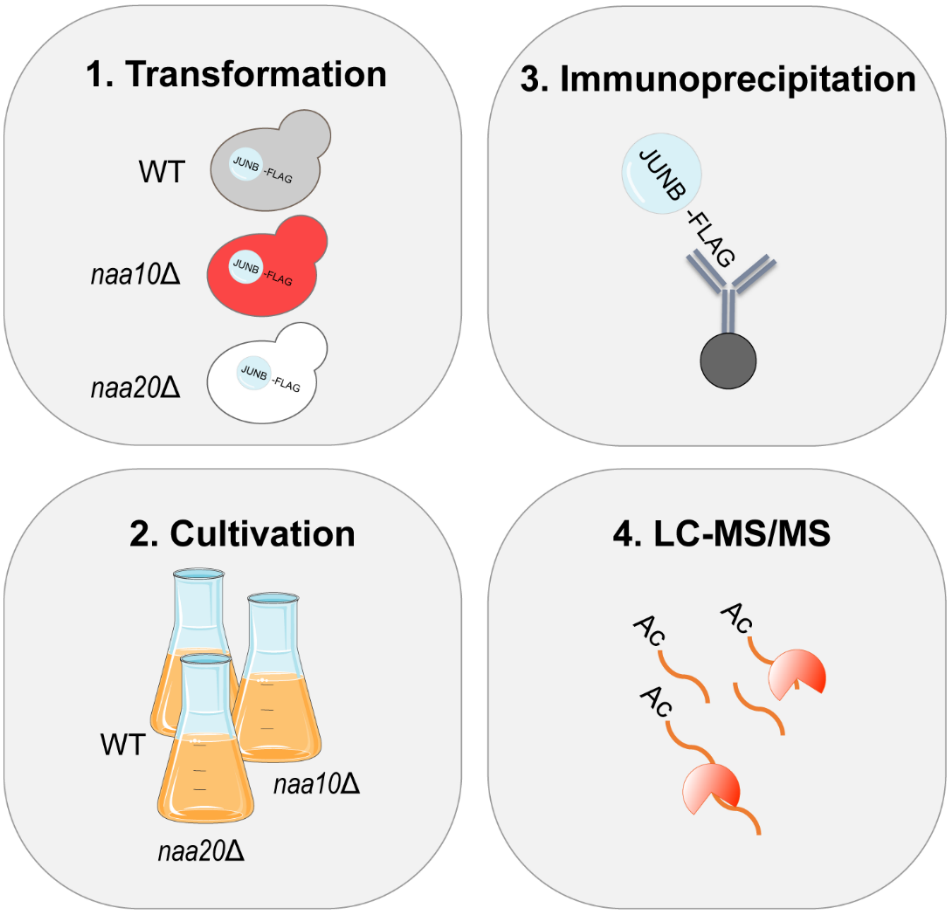

Supplementary Figure 7: Workflow for assessing human JUNB Nt Cys acetylation using budding yeast and LC-MS/MS

Schematic workflow of JUNB-FLAG yeast transformation followed by LC-MS/MS analysis. Three *S. cerevisiae* strains WT, *naa10Δ* and *naa20Δ* with the last two lacking functional NatA and NatB respectively, were transformed with a JUNB-FLAG plasmid or control plasmid (1). After transformation the strains were cultured (2) and harvested for immunoprecipitation (IP) (3). The IP-samples were further digested with a protease before peptide analysis by LC-MS/MS (4). Figure was made using images from Servier Medical Art, licensed under a Creative Commons Attribution 4.0 Unported License

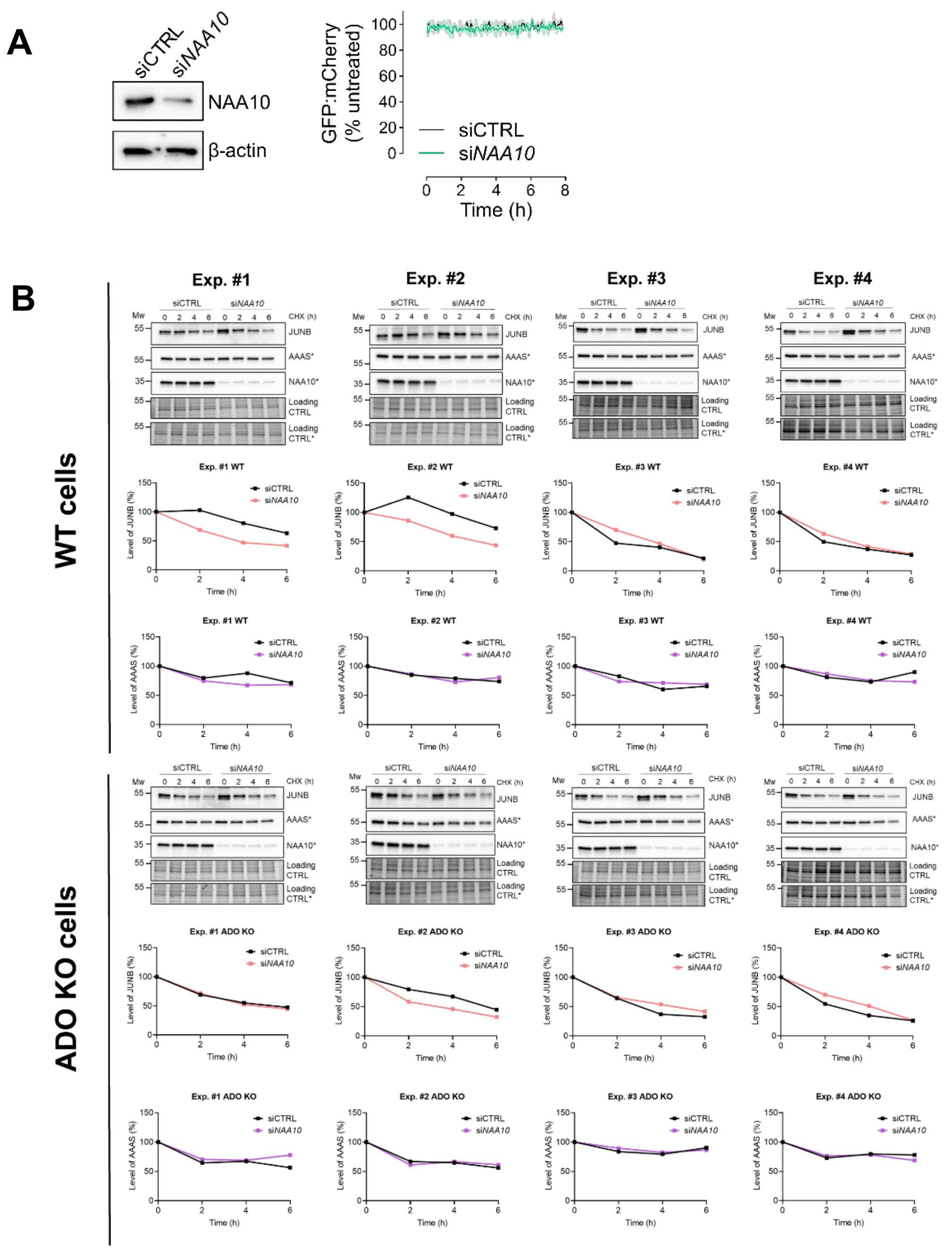

Supplementary Figure 8. Cycloheximide pulse-chase experiments to investigate stability of cellular Nt-Cys substrates.

A. **RGS4[K3D]-DFOR remained stable in siCTRL and si*NAA10* treated SH-SY5Y cells.** SH-SY5Y cells stably expressing $RGS4_{1\text{-}15}$[K3D]-DFOR were transfected with either control siRNA (siCTRL) or siRNA targeting *NAA10* (si*NAA10*). 72h later cells were treated with 50 µg/mL cycloheximide (CHX) and the fluorescence of GFP and mCherry monitored for 8 hours. The ratio of GFP to mCherry fluorescence (GFP:mCherry) is expressed relative to control cells (without CHX treatment). A representative immunoblot demonstrating knock-down of NAA10 protein levels in parallel cells. Data represent the mean ± S.D. from 3 independent experiments.

B. JUNB and AAAS remained stable in siCTRL and si*NAA10* treated HAP1 WT and HAP1 ADO KO cells. Cells were transfected with either siCTRL or si*NAA10* 24 h after seeding. 72 h post transfection cells were treated with 50 µg/mL CHX. Western blotting (WB) was used to determine the levels of JUNB and AAAS in a 0-6 h time course. Gel image was used as a loading control and anti-NAA10 putative NatA formation. Stability diagrams showing the total amount of JUNB and AAAS in siCTRL or si*NAA10* treated cells, where the levels of JUNB (red) and AAAS (purple) were adjusted in relation to the loading control for uniform protein input, and the amount of protein present at 0 h. The blots represent four independent (n=4) setups with their corresponding graphs. Blots with an asterisk (*) coincide.

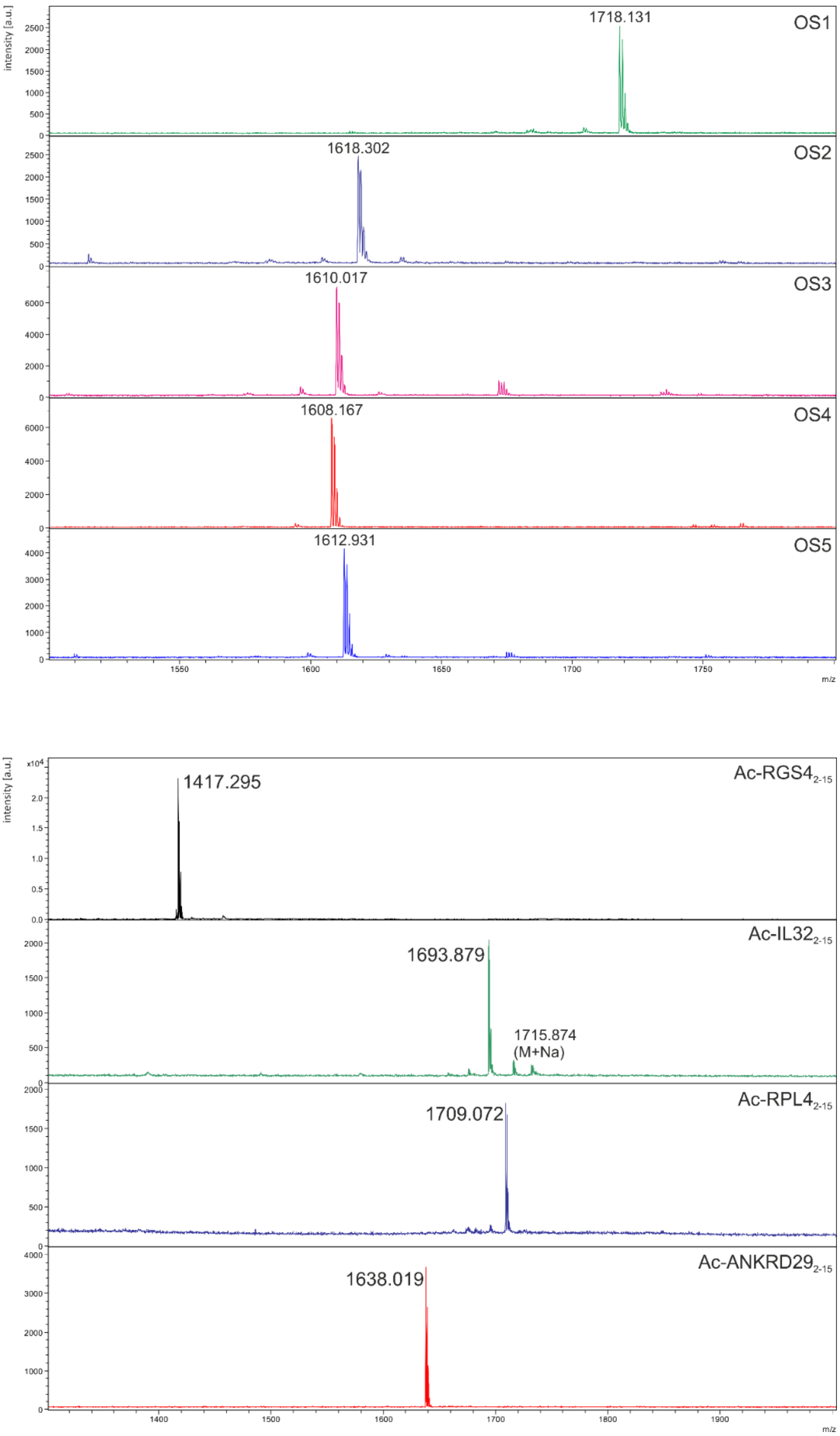

Supplementary Figure 9: Matrix-assisted laser desorption/ionization-time of flight mass spectra spectra of synthesised peptides

Expected molecular weights: OS1 = 1717.09; OS2 = 1617.95; OS3 = 1608.94; OS4 = 1611.95; OS5 = 1607.97; Ac-RGS4$_{2\text{-}15}$ = 1416.73; Ac-IL32$_{2\text{-}15}$ = 1693.10; Ac-RPL4$_{2\text{-}15}$ = 1708.04; Ac-ANKRD29$_{2\text{-}15}$ = 1636.95.

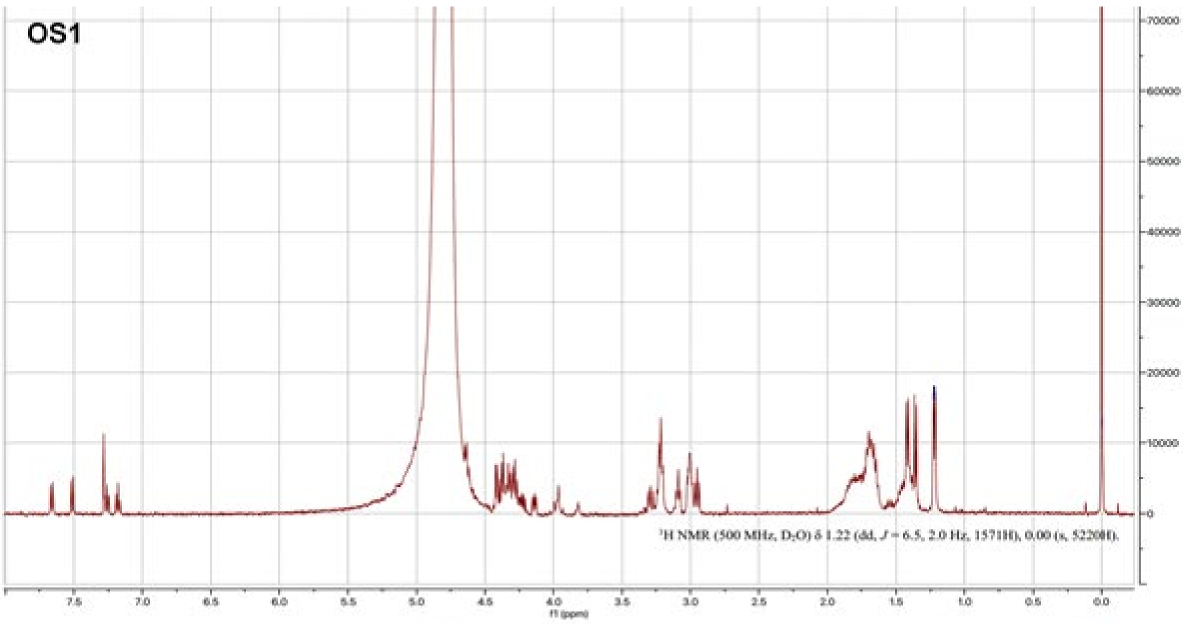
OS1
^{1}H NMR (500 MHz, D_2O) δ 1.22 (dd, *J* = 6.5, 2.0 Hz, 1571H), 0.00 (s, 5220H).
f1 (ppm)

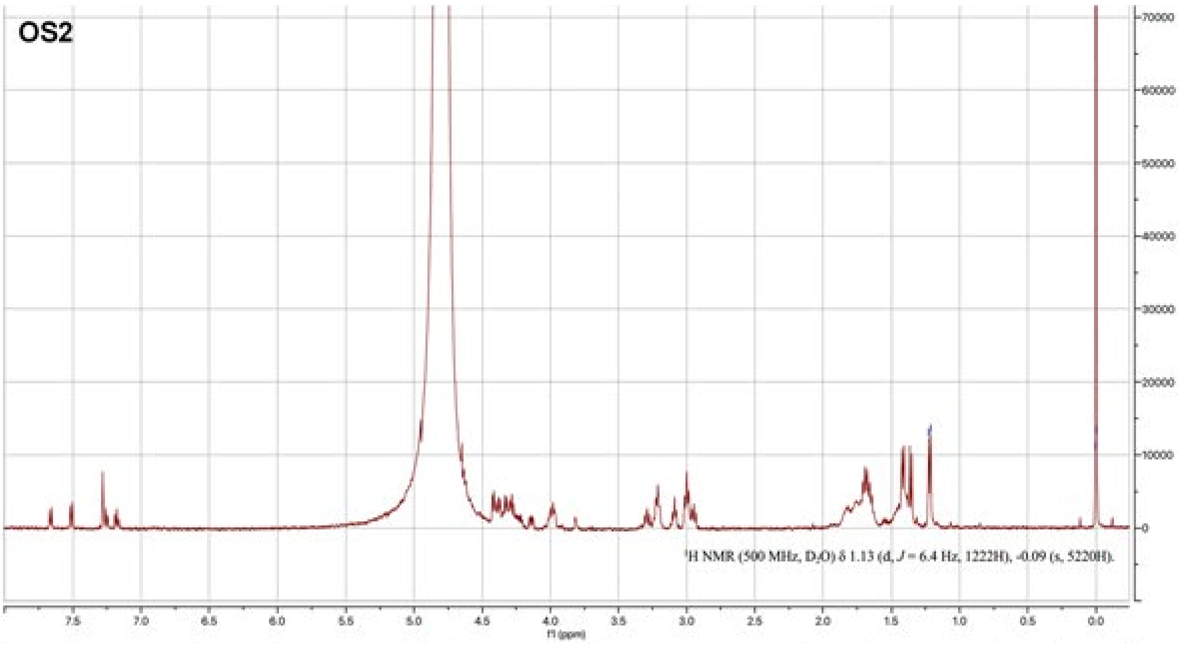
OS2
^{1}H NMR (500 MHz, D_2O) δ 1.13 (d, *J* = 6.4 Hz, 1222H), -0.09 (s, 5220H).
f1 (ppm)

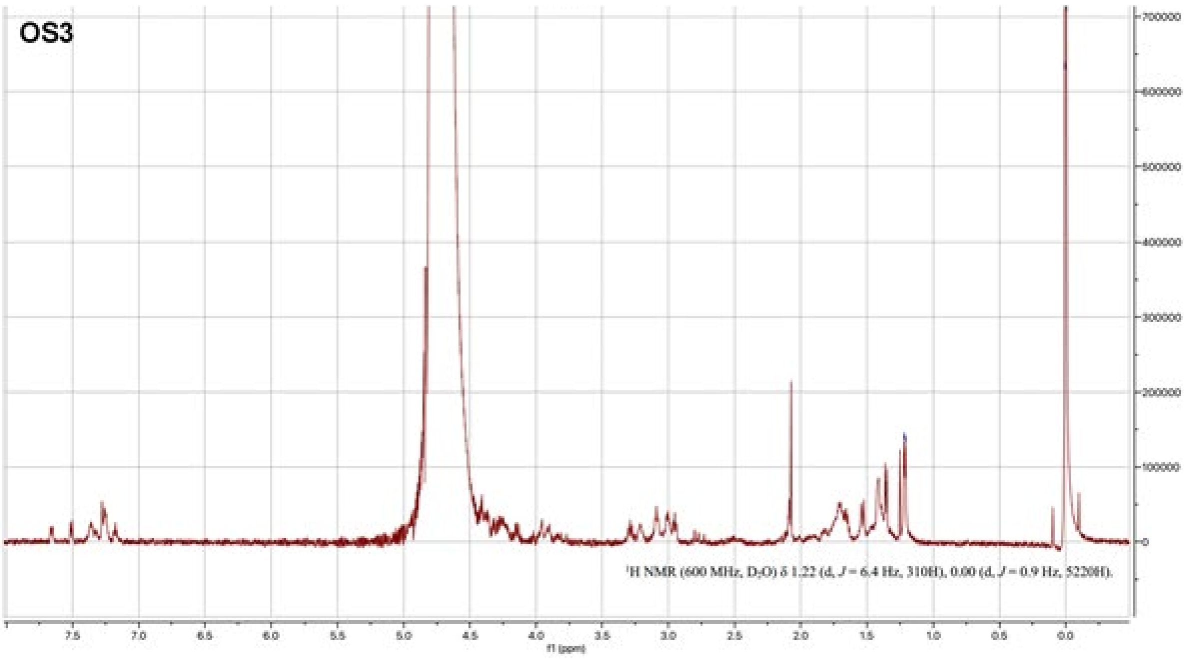
OS3
^{1}H NMR (600 MHz, D_2O) δ 1.22 (d, *J* = 6.4 Hz, 310H), 0.00 (d, *J* = 0.9 Hz, 5220H).
f1 (ppm)

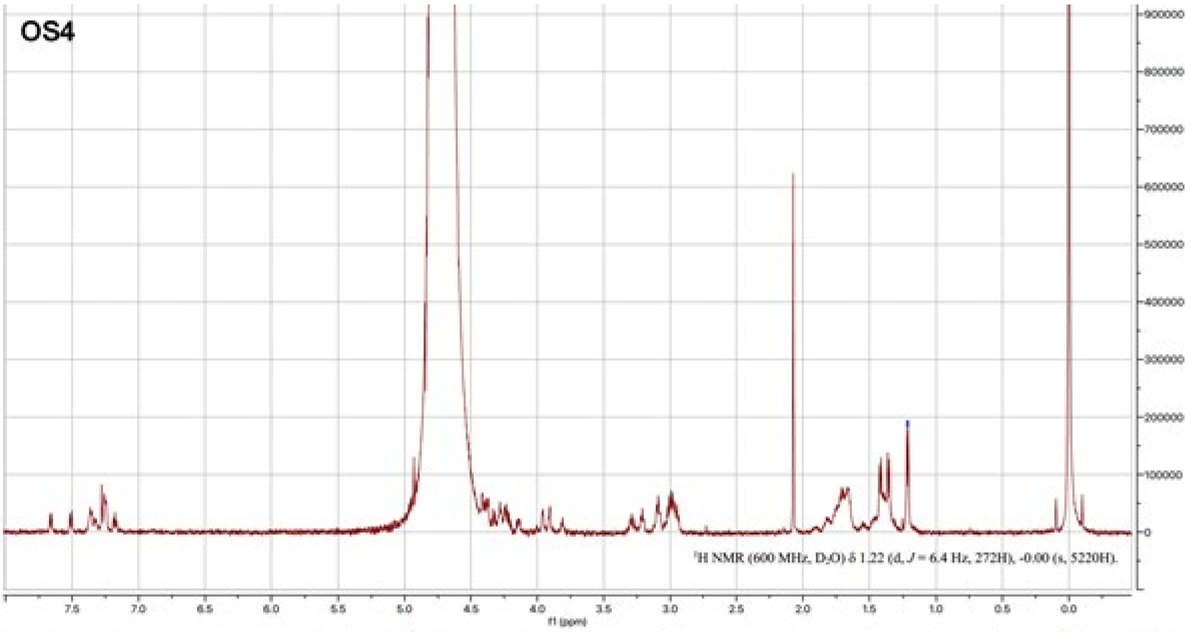

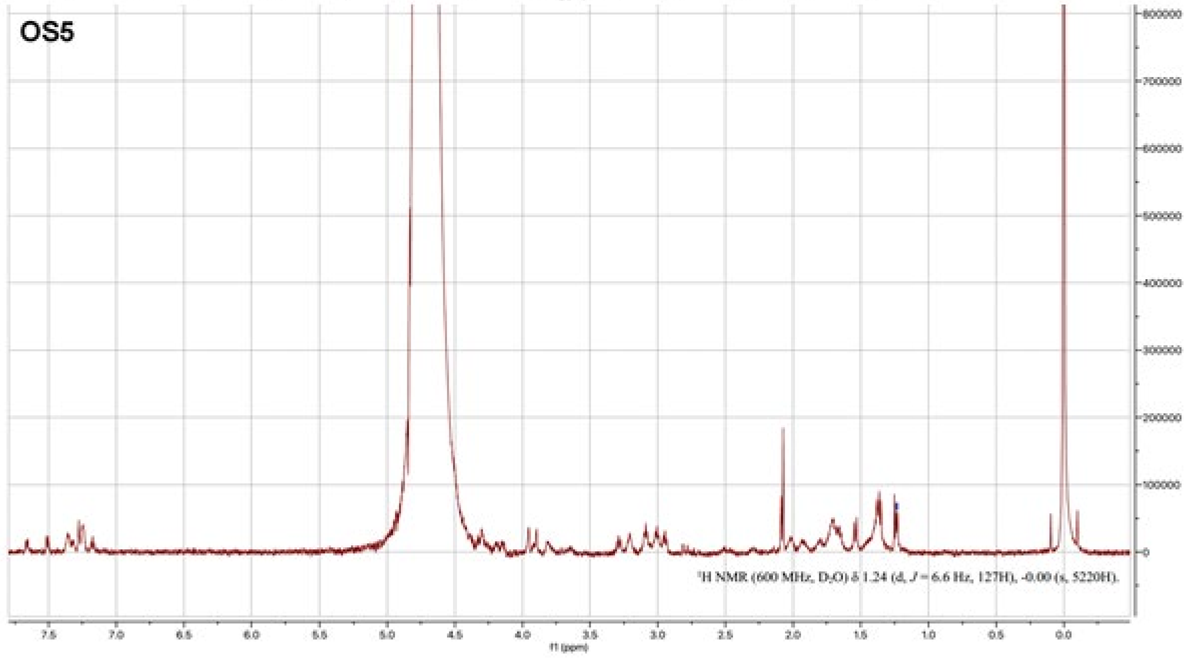

Supplementary Figure 10: Quantification of OS1-5 synthesised peptides by NMR

The concentration of peptide in each sample was calculated using the following formula:

$$C_x = \frac{I_x}{I_{cal}} \times \frac{N_{cal}}{N_x} \times C_{cal}$$

Where I, N and C are the integral area, number of protons and concentration of the peptide (x) and calibrant (cal), respectively. The calibrant used was 3-(trimethylsilyl)propionic-2,2,3,3-d_4 acid (0.00 ppm): $C_{cal} = 58\ mM$; $N_{cal} = 9$; I_x was set to 5220 in each spectrum. The ^{1}H NMR peaks for the threonine methyl groups in the peptide were used in each case. In each spectrum, multiplets are reported for relevant calibrant and peptide peaks. OS1: 50 mM expected, 26.2 mM calculated. OS2: 50 mM expected, 20.4 mM calculated. OS3: 10 mM expected, 4.54 mM calculated. OS4: 10 mM expected, 4.22 mM calculated. OS5: 10 mM expected, 5.16 mM calculated. For OS peptide sequences, see Figure 2.

Accession	Gene Name	Peptide Sequence	Initial Activity %
P63167	***DYNLL1***	C D R K A V I R W G R P V G R R R R P V R V Y P	119.52
P17096	***HMGA1***	S E S S S K S R W G R P V G R R R R P V R V Y P	100
O60678	***PRMT3***	C S L A S G A R W G R P V G R R R R P V R V Y P	32.49
Q9NRG9	***AAAS***	C S L G L F P R W G R P V G R R R R P V R V Y P	23.34
P58546	***MTPN***	C D K E F M W R W G R P V G R R R R P V R V Y P	22.55
P17275	***JUNB***	C T K M E Q P R W G R P V G R R R R P V R V Y P	22.19
P46695	***IER3/IEX1***	C H S R S C H R W G R P V G R R R R P V R V Y P	10.23
B4DNV3	***IL6***	C E S S K E A R W G R P V G R R R R P V R V Y P	8.11
P78549	***NHTL1***	C S P Q E S G R W G R P V G R R R R P V R V Y P	7.59
P60660	***MYL6***	C D F T E D Q R W G R P V G R R R R P V R V Y P	6.71
Q8IXN7	***RIMKLA***	C S Q L W F L R W G R P V G R R R R P V R V Y P	6.04
Q8N8J7	***FAM241A***	C S A G E L L R W G R P V G R R R R P V R V Y P	5.50
P62736	***ACTA2***	C E E E D S T R W G R P V G R R R R P V R V Y P	3.38
Q8TBG4	***ETNPPL***	C E L Y S K R R W G R P V G R R R R P V R V Y P	3.30
P07203	***GPX1***	C A A R L A A R W G R P V G R R R R P V R V Y P	1.01
O94808	***GFPT2***	C G I F A Y M R W G R P V G R R R R P V R V Y P	0.88
Q9Y277	***VDAC3***	C N T P T Y C R W G R P V G R R R R P V R V Y P	0.74
Q13077	***TRAF1***	C Y R A P C S R W G R P V G R R R R P V R V Y P	0.57
O15492	***RGS16***	C R T L A A F R W G R P V G R R R R P V R V Y P	0.49
P24001	***IL32***	C F P K V L S R W G R P V G R R R R P V R V Y P	0.48
P21589	***NT5E***	C P R A A R A R W G R P V G R R R R P V R V Y P	0.26
Q60I27	***ALS2CL***	C N P E E A A R W G R P V G R R R R P V R V Y P	0.26
P08243	***CHURC1***	C G D C V E K R W G R P V G R R R R P V R V Y P	0.25
O95372	***LYPLA2***	C G N T M S V R W G R P V G R R R R P V R V Y P	0.17
O15547	***P2RX6***	C P Q L A G A R W G R P V G R R R R P V R V Y P	0.16
Q92537	***SUSD6***	C H G R I A P R W G R P V G R R R R P V R V Y P	0.15
Q15465	***SHHC***	C F P G S A T R W G R P V G R R R R P V R V Y P	0.15
P49798	***RGS4***	C K G L A G L R W G R P V G R R R R P V R V Y P	0.13
A6NNY8	***USP27X***	C K D Y V Y D R W G R P V G R R R R P V R V Y P	0.04
Q5JW98	***CAHLM4***	C P T L N N I R W G R P V G R R R R P V R V Y P	0.03
Q8N6D5	***ANKRD29***	C R M S F K K R W G R P V G R R R R P V R V Y P	0.00
Q8WUH1	***ASNS***	C G I W A L F R W G R P V G R R R R P V R V Y P	0.00
P19022	***CDH2***	C R I A G A L R W G R P V G R R R R P V R V Y P	0.00
	OS7	C F G R R M K R W G R P V G R R R R P V R V Y P	0.00
Q504Y0	***ZIP12***	C F R T K L S R W G R P V G R R R R P V R V Y P	0.00

Supplementary Table 1: Full *in vitro* NatA substrate screening data

Protein	UniProt ID	Nt-Sequence	Nt-Ac	Peptide ID start	Cell line*	References
MYL6	P60660	(M)CDFTE	100%	2	a,b,c,d,e	[6-11]
F241A	Q8N8J7	(M)CSAGE	100%	2	d	[10]
PRMT3/ANM3	O60678	(M)CSLAS	100%	2	a,b,c,d	[7-10,12]
Aladin/AAAS	Q9NRG9	(M)CSLGL	100%	2	a,c,d	[8,9,12]
NTH/NTHL1	P78549	(M)CSPQE	100%	2	a	[8]
JUNB	P17275	(M)CTKME	100%	2	b,d	[9,10]
MTPN	P58546	(M)CDKEF	0/100%	2	a (100%), c (0%)	[7,9]
CHURC1	Q8WUH1	(M)CGDCV	0%	2	a,b,c,d	[7-10,12]
SMO	Q99835	CAIVER	100%	154	d	[12]
IST1	P53990	CDLLLA	100%	76	a	[8]
GNPTA	Q3T906	CSGNSG	100%	468	c	[9]

Supplementary Table 2: Cysteine-starting N-termini and their acetylation status in human cells defined by targeted mass spectrometry.

*Cell lines: a, HeLa; b, B-cells; c, fibroblasts; d, A431; e, HAP1

Name	NatA	ADO
	5 min	1 min
	Acetylation normalised to SESS (%)	Oxidation normalised to RGS4 (%)
OS7	-0.41	152.16
IL32	0.48	110.79
ANKRD29	-0.24	103.04
RGS4	0.13	100.00
SUSD6	0.15	97.75
CDH2	-0.25	55.80
IER3/IEX1	10.23	48.36
GPX1	1.01	42.05
VDAC3	0.74	27.79
PRMT3	32.49	24.87
TRAF1	0.57	24.36
RGS16	0.49	22.61
RIMKLA	6.04	22.50
AAAS	23.34	19.24
ZIP12	-0.06	18.14
SHHC	0.15	18.11
ETNPPL	3.30	13.77
DYNLL1	119.52	11.31
FAM241A	5.50	9.59
GFPT2	0.88	7.78
NTHL1	7.59	7.41
NT5E	0.26	6.62
JUNB	22.19	4.92
CAHLM4	0.03	4.47
USP27X	0.04	4.12
MTPN	22.55	3.72
ALS2CL	0.26	3.30
IL6	8.11	2.82
P2RX6	0.16	2.74
MYL6	6.71	1.91
LYPLA2	0.17	0.96
CHURC1	0.25	0.00
ASNS	-0.06	-0.03
ACTA2	3.38	14mer peptide insoluble
HMGA1	100	

Supplementary Table 3: Combined *in vitro* data for Nt-Cys sequences

Ac-RGS4	Ac-CKGLAGLPASCLRS-NH_2
Ac-IL32	Ac-CFPKVLSDDMKKLK-NH_2
Ac-RPL4	Ac- CRGGRMFAPTKTWR-NH_2
Ac-ANKRD29	Ac- CRMSFKKETPLANA-NH_2
OS1	CRGRRKKATTKAWR-NH_2
OS2	CRGGRKKATTKAWR-NH_2
OS3	CRGGFKKATTKAWR-NH_2
OS4	CRGGFMKATTKAWR-NH_2
OS5	CRGGFMKAPTKAWR-NH_2

Supplementary Table 4: Peptides synthesised by Solid-Phase Peptide Synthesis

Primer Name	Sequence
For_HindIII_RGS4	ATTAAGCTTGCCACCATGTGCAAAGGCCTGGCAGGCCTGCCAGCAAGCTGCCTGAGGAGC
For_HindIII_AAAS	ATTAAGCTTGCCACCATGTGCAGCCTGGGCCTGTTCCCACCACCACCACCAAGGGGCCAA
For_HindIII_ANKRD29	ATTAAGCTTGCCACCATGTGCAGGATGAGCTTCAAGAAGGAGACCCCCCTGGCCAACGCC
For_HindIII_ASNS	ATTAAGCTTGCCACCATGTGCGGAATCTGGGCCCTGTTCGGCAGCGACGACTGCCTGAGC
For_HindIII_At RAP 2.12	ATTAAGCTTGCCACCATGTGCGGAGGAGCAATCATCAGCGACTTCATCCCCCCCCCCAGG
For_HindIII_GFPT2	ATTAAGCTTGCCACCATGTGCGGCATCTTCGCCTACATGAACTACAGGGTGCCCAGGACC
For_HindIII_GPX1	ATTAAGCTTGCCACCATGTGCGCAGCAAGGCTGGCAGCAGCAGCAGCAGCAGCACAGAGC
For_HindIII_JUNB	ATTAAGCTTGCCACCATGTGCACCAAGATGGAGCAGCCCTTCTACCACGACGACAGCTAC
For_HindIII_LYPLA2	ATTAAGCTTGCCACCATGTGCGGCAACACCATGAGCGTGCCCCTGCTGACCGACGCAGCA
For_HindIII_MTPN	ATTAAGCTTGCCACCATGTGCGACAAGGAGTTCATGTGGGCCCTGAAGAACGGCGACCTG
For_HindIII_OS7	ATTAAGCTTGCCACCATGTGCTTCGGCAGGAGGATGAAGGCCACCACCAAGGCCTGGAGG
For_HindIII_SUSD6	ATTAAGCTTGCCACCATGTGCCACGGCAGGATCGCACCCAAGAGCACCAGCGTGTTCGCA
Rev_GFP_XbaI	GCCTCTAGAGCCCTTGTACAGCTCGTCCA

Supplementary Table 5: Primers used to generate MCX13-GFP constructs for the DFOR assay.

Supplementary References

1. White, M. D. *et al.* Structures of *Arabidopsis thaliana* oxygen-sensing plant cysteine oxidases 4 and 5 enable targeted manipulation of their activity. *Proceedings of the National Academy of Sciences* **117**, 23140–23147 (2020).

2. Fernandez, R. L. *et al.* The Crystal Structure of Cysteamine Dioxygenase Reveals the Origin of the Large Substrate Scope of This Vital Mammalian Enzyme. *Biochemistry* **60**, 3728–3737 (2021).

3. Wang, Y., Shin, I., Li, J. & Liu, A. Crystal structure of human cysteamine dioxygenase provides a structural rationale for its function as an oxygen sensor. *J Biol Chem* **297**, 101176 (2021).

4. Gunawardana, D. M., Heathcote, K. C. & Flashman, E. Emerging roles for thiol dioxygenases as oxygen sensors. *The FEBS Journal* **289**, 5426–5439 (2022).

5. Smith, E. & Keeley, T. P. Monitoring ADO dependent proteolysis in cells using fluorescent reporter proteins. *Methods Enzymol* **686,** 267–295 (2023).

6. Arnesen, T. *et al.* Proteomics analyses reveal the evolutionary conservation and divergence of N-terminal acetyltransferases from yeast and humans. *Proc Natl Acad Sci USA* **106**, 8157-62 (2009).

7. Van Damme P *et al.* N-terminal acetylome analyses and functional insights of the N-terminal acetyltransferase NatB. *PLoS Genet* **7**, e1002169 (2011).

8. Van Damme P *et al.* NatF contributes to an evolutionary shift in protein N-terminal acetylation and is important for normal chromosome segregation. *Proc Natl Acad Sci USA* **109**, 12449-54 (2012).

9. Myklebust L *et al.* Biochemical and cellular analysis of Ogden syndrome reveals downstream Nt-acetylation defects *Hum Mol Genet* **24,** 1956-76 (2015).

10. Van Damme P *et al.* A Role for Human N-alpha Acetyltransferase 30 (Naa30) in Maintaining Mitochondrial Integrity. *Mol Cell Proteomics* **15,** 3361-72 (2016).

11. Drazic A *et al.* NAA80 is actin's N-terminal acetyltransferase and regulates cytoskeleton assembly and cell motility. *Proc Natl Acad Sci USA* **115**, 4399-4404 (2018).

12. Aksnes H *et al.* An organellar nα-acetyltransferase, naa60, acetylates cytosolic N termini of transmembrane proteins and maintains Golgi integrity. *Cell Rep* **10**, 1362-74 (2015).
