# Supplementary material for: N-terminal cysteine acetylation and oxidation patterns may define protein stability
Source: Nat Commun. 2024 Jun 25;15:5360. doi: 10.1038/s41467-024-49489-2 (PMC11199558; doi:10.1038/s41467-024-49489-2)
Supplement: Supplementary file 3 — Description of additional supplementary files [file 41467_2024_49489_MOESM3_ESM.pdf]

Description of Additional Supplementary Files

Supplementary Data 1. Supplementary Tables 1-5.

Supplementary Data 2. Full in vitro substrate screening data.

Supplementary Data 3. One-Way ANOVA followed by Tukeys's Multiple Comparison Test of OS Peptide 1 minute Screening Data.

Supplementary Data 4. One-Way ANOVA followed by Tukeys's Multiple Comparison Test of AS Peptide 1 minute Screening Data.

Supplementary Data 5. Fold changes observed in DFOR assays in Figure 4.
