# Supplementary material for: N-terminal cysteine acetylation and oxidation patterns may define protein stability
Source: Nat Commun. 2024 Jun 25;15:5360. doi: 10.1038/s41467-024-49489-2 (PMC11199558; doi:10.1038/s41467-024-49489-2)
Supplement: Supplementary file 9 — Reporting Summary [file 41467_2024_49489_MOESM9_ESM.pdf]

nature portfolio

Corresponding author(s): Emily Flashman

Last updated by author(s): Mar 28, 2024

Reporting Summary

Nature Portfolio wishes to improve the reproducibility of the work that we publish. This form provides structure for consistency and transparency in reporting. For further information on Nature Portfolio policies, see our Editorial Policies and the Editorial Policy Checklist.

Statistics

For all statistical analyses, confirm that the following items are present in the figure legend, table legend, main text, or Methods section.

n/a	Confirmed	
☐	☒	The exact sample size (*n*) for each experimental group/condition, given as a discrete number and unit of measurement
☐	☒	A statement on whether measurements were taken from distinct samples or whether the same sample was measured repeatedly
☐	☒	The statistical test(s) used AND whether they are one- or two-sided<br>*Only common tests should be described solely by name; describe more complex techniques in the Methods section.*
☒	☐	A description of all covariates tested
☒	☐	A description of any assumptions or corrections, such as tests of normality and adjustment for multiple comparisons
☐	☒	A full description of the statistical parameters including central tendency (e.g. means) or other basic estimates (e.g. regression coefficient) AND variation (e.g. standard deviation) or associated estimates of uncertainty (e.g. confidence intervals)
☐	☒	For null hypothesis testing, the test statistic (e.g. *F*, *t*, *r*) with confidence intervals, effect sizes, degrees of freedom and *P* value noted<br>*Give P values as exact values whenever suitable.*
☒	☐	For Bayesian analysis, information on the choice of priors and Markov chain Monte Carlo settings
☒	☐	For hierarchical and complex designs, identification of the appropriate level for tests and full reporting of outcomes
☒	☐	Estimates of effect sizes (e.g. Cohen's *d*, Pearson's *r*), indicating how they were calculated

Our web collection on statistics for biologists contains articles on many of the points above.

Software and code

Policy information about availability of computer code

Data collection	LC-MS/MS analysis of immunoprecipitated JunB-FLAG: Mass spectrometric data were acquired using the Orbitrap Eclipse mass spectrometer. Instrument control as through Orbitrap Eclipse Tune 3.5/3.1 and Xcalibur 4.5/4.4.<br>LC-MS analysis of peptides tested in ADO/NatA activity assays: Peptide oxidation was measured by high-throughput MS using a RapidFire RF360 sampling robot (Agilent) connected to an Agilent 6530 Accurate-Mass Q-ToF mass spectrometer. Spectra were assessed in MassHunter Qualitative Analysis B.07.00 (Agilent).<br>DFOR assay: GFP and mCherry fluorescence were measured by multichromatic fluorescence imaging using a FLUOstar (BMG Lab-Tech)
Data analysis	LC-MS analysis of peptides tested in ADO/NatA activity assays: data were analysed using MassHunter Qualitative Analysis software vB.07.00.<br>Peptide oxidation analysis was conducted using the RapidFire Integrator software (Agilent).<br>LC-MS/MS analysis of immunoprecipitated JunB-FLAG: proteomics data were analysed using Proteome Discoverer.<br>Graphical data were prepared and statistical analyses conducted using GraphPad Prism v10.

For manuscripts utilizing custom algorithms or software that are central to the research but not yet described in published literature, software must be made available to editors and reviewers. We strongly encourage code deposition in a community repository (e.g. GitHub). See the Nature Portfolio guidelines for submitting code & software for further information.

Data

Policy information about availability of data

All manuscripts must include a data availability statement. This statement should provide the following information, where applicable:

- Accession codes, unique identifiers, or web links for publicly available datasets
- A description of any restrictions on data availability
- For clinical datasets or third party data, please ensure that the statement adheres to our policy

The authors confirm that all data supporting the findings presented in this study are available in the article and its supplemental data files, except for mass spectrometry proteomics data, which have been deposited to the ProteomeXchange Consortion (http://proteomecentral.proteomexchanges.org) via the PRIDE partner repository. Details for retrieving this data are provided in the Source Data files and Data Availability Statement. PDB 7LVZ has been cited and used in this study and links to access this data provided in the Data Availability Statement.

Research involving human participants, their data, or biological material

Policy information about studies with human participants or human data. See also policy information about sex, gender (identity/presentation), and sexual orientation and race, ethnicity and racism.

Reporting on sex and gender	n/a
Reporting on race, ethnicity, or other socially relevant groupings	n/a
Population characteristics	n/a
Recruitment	n/a
Ethics oversight	n/a

Note that full information on the approval of the study protocol must also be provided in the manuscript.

Field-specific reporting

Please select the one below that is the best fit for your research. If you are not sure, read the appropriate sections before making your selection.

☒ Life sciences ☐ Behavioural & social sciences ☐ Ecological, evolutionary & environmental sciences

For a reference copy of the document with all sections, see nature.com/documents/nr-reporting-summary-flat.pdf

Life sciences study design

All studies must disclose on these points even when the disclosure is negative.

Sample size	Sample size is indicated in the Figure legend and/or in the Methods. No statistical method was used to predetermine sample size given we were not analysing large cohorts (predominantly biochemical study rather than analysing biological cohorts).
Data exclusions	All technically sound data were included.
Replication	Experiments were replicated for the indicated number of times, at least three times for most peptide and cell culture experiments. LC-MS/MS analysis of immunoprecipitated JUNB-FLAG was performed using two (HAP1) or three (yeast cells) independent samples per condition. All attempts to replicate the data here were successful.
Randomization	No randomisation was performed in this study as data was not derived from differently treated groups.
Blinding	For mass spectrometric analysis, blinding was not done since the sample groups have to be identified during data analysis. Determination of significance is solely based on automated procedures. For immunoblotting blinding was not done since data was not derived from different treatment groups and samples were prepared and analysed by the same researcher in small batches.

Reporting for specific materials, systems and methods

We require information from authors about some types of materials, experimental systems and methods used in many studies. Here, indicate whether each material, system or method listed is relevant to your study. If you are not sure if a list item applies to your research, read the appropriate section before selecting a response.

Materials & experimental systems

n/a	Involved in the study
☐	☒ Antibodies
☐	☒ Eukaryotic cell lines
☒	☐ Palaeontology and archaeology
☒	☐ Animals and other organisms
☒	☐ Clinical data
☒	☐ Dual use research of concern
☒	☐ Plants

Methods

n/a	Involved in the study
☒	☐ ChIP-seq
☒	☐ Flow cytometry
☒	☐ MRI-based neuroimaging

Antibodies

Antibodies used

AAAS - Santa Cruz Biotechnology, Catalogue number: sc-374073 (1:1000)
ASNS - Santa Cruz Biotechnology, Catalogue number: sc-3765809 (1:1000)
JunB - Cell Signalling Technology, Catalogue number: 3753, or Abcam, Catalogue number: Ab128878, Lot: GR3263077-2 (1:1000)
ADO - Abcam, Catalogue number: ab134102 (1:1000)
LYPLA2 - Santa Cruz Biotechnology, Catalogue number: sc-390546 (1:1000)
GPX-1 - Cell Signalling Technology, Catalogue number: 3286 (1:500)
MTPN - Santa Cruz Biotechnology, Catalogue number: sc-166072 (1:500)
RGS4 - Cell Signaling Technology, Catalogue number: 15129 (1:1000)
RGS5 - Santa Cruz Biotechnology, Catalogue number: sc-514184 (1:500)
Beta-actin HRP - Abcam, Catalogue number: ab49900 (1:25000)
GFPT2 - Novus Biologicals, Catalogue number: NBP1-56688 (1:1000)
ANKRD29 - Proteintech, Catalogue number: 23999-1-AP (1:1000)
SUSD6 - Novus Biologicals, Catalogue number: H00009766-801P (1:1000)
Flag-HRP (M2) - SigmaAldrich, Catalogue number: A8592 (1:1000)
NAA10 - Cell Signalling, Catalogue number: 13357, Lot: 1 (1:2000)
NAA20 - Sigma Aldrich, Catalogue number: HPA063344, Lot: R88139. (1:1000)
These are all listed in the methods section of the manuscript and/or source data file

Validation

All antibodies are described in Supplemental Information and Source data files. Antibodies were used only if they (i) detected a prominent product that migrated at the expected size for the protein in question by SDS-PAGE, and (ii) detected the intended target in HEK-293T cells transiently transfected with the desired gene, and not in the untransfected control.

Eukaryotic cell lines

Policy information about cell lines and Sex and Gender in Research

Cell line source(s)

SH-SY5Y -ECACC 94030304, human, female
HEK-293T - ATCC CRL-3216, human, female
U87-MG - ECACC 89091402, human, female
The near haploid human HAP1 WT cells (clone C631; sex: male; RRID:CVCL Y019) and the HAP1 gene-KO cell line ADO KO cells (HZGHC00812c004) were obtained from Horizon Discovery Ltd in Cambridge, United Kingdom.

Authentication

SH-SY5Y cells were authenticated by STR profiling. HEK-293T , HAP1 and U-87 MG cells were not authenticated. The ADO KO cells were confirmed by Sanger sequencing of PCR products of the edited genomic region as well as Western blotting using anti-ADO (Abcam, Catalogue number: Ab134102).

Mycoplasma contamination

All cell cultures were tested for mycoplasma contamination on a monthly basis using the MycoAlert Detection Kit (Lonza) or by NucBlue DAPI staining (Invitrogen) and all data reported here were obtained in cultures that tested negative.

Commonly misidentified lines
(See ICLAC register)

No commonly misidentified lines were used.

Plants

Seed stocks

Report on the source of all seed stocks or other plant material used. If applicable, state the seed stock centre and catalogue number. If plant specimens were collected from the field, describe the collection location, date and sampling procedures.

Novel plant genotypes

Describe the methods by which all novel plant genotypes were produced. This includes those generated by transgenic approaches, gene editing, chemical/radiation-based mutagenesis and hybridization. For transgenic lines, describe the transformation method, the number of independent lines analyzed and the generation upon which experiments were performed. For gene-edited lines, describe the editor used, the endogenous sequence targeted for editing, the targeting guide RNA sequence (if applicable) and how the editor was applied.

Authentication

Describe any authentication procedures for each seed stock used or novel genotype generated. Describe any experiments used to assess the effect of a mutation and, where applicable, how potential secondary effects (e.g. second site T-DNA insertions, mosiacism, off-target gene editing) were examined.
