# Supplementary material for: N-terminal cysteine acetylation and oxidation patterns may define protein stability
Source: Nat Commun. 2024 Jun 25;15:5360. doi: 10.1038/s41467-024-49489-2 (PMC11199558; doi:10.1038/s41467-024-49489-2)
Supplement: Supplementary file 10 — Source Data [file 41467_2024_49489_MOESM10_ESM.zip › NCOMMS-23-38359 Source Data/Figure 8B Source Data.pptx]

## Slide 1
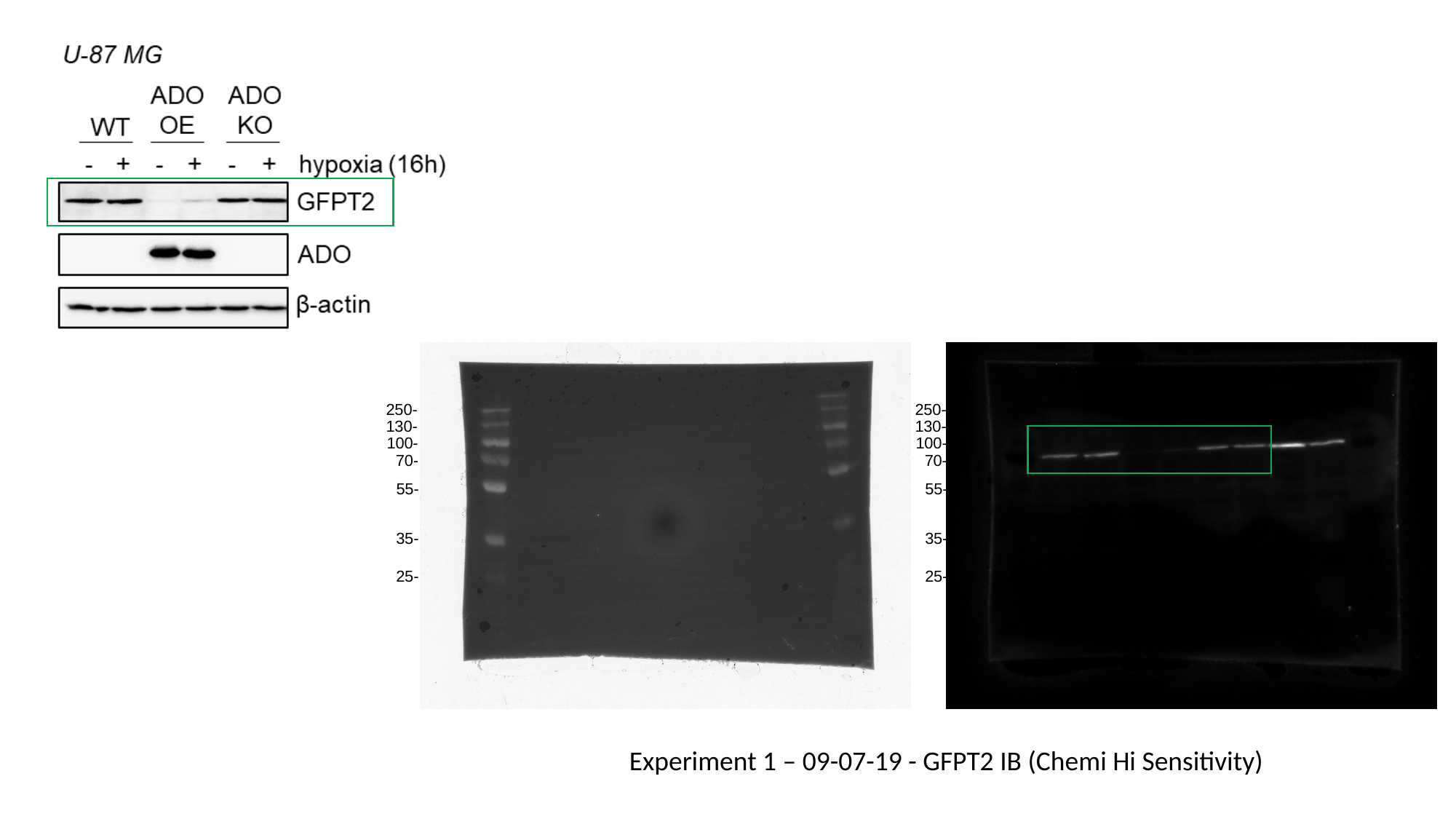

250-
250-
130-
130-
100-
100-
70-
70-
55-
55-
35-
35-
25-
25-
Experiment 1 – 09-07-19 - GFPT2 IB (Chemi Hi Sensitivity)

## Slide 2
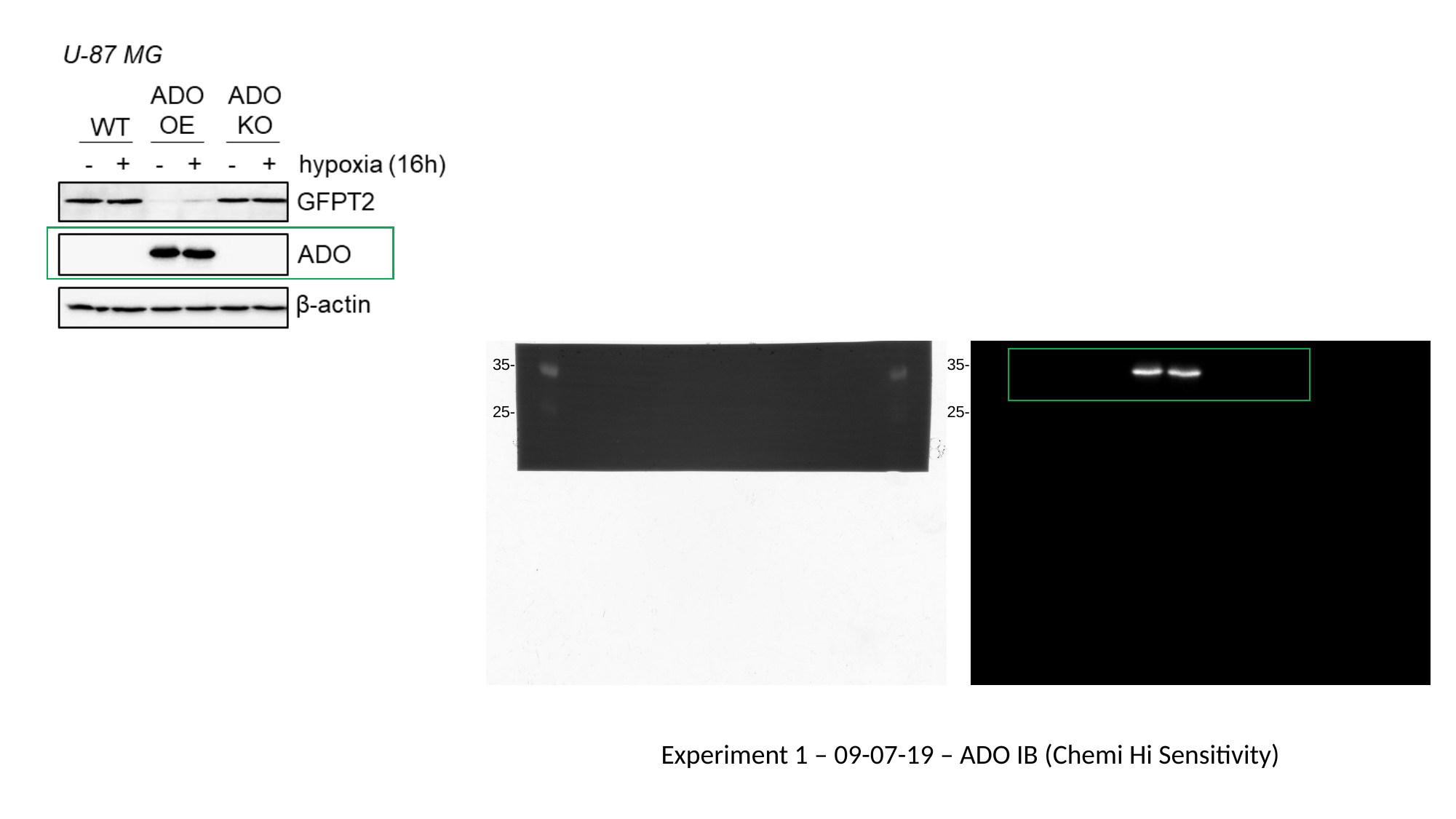

35-
35-
25-
25-
Experiment 1 – 09-07-19 – ADO IB (Chemi Hi Sensitivity)

## Slide 3
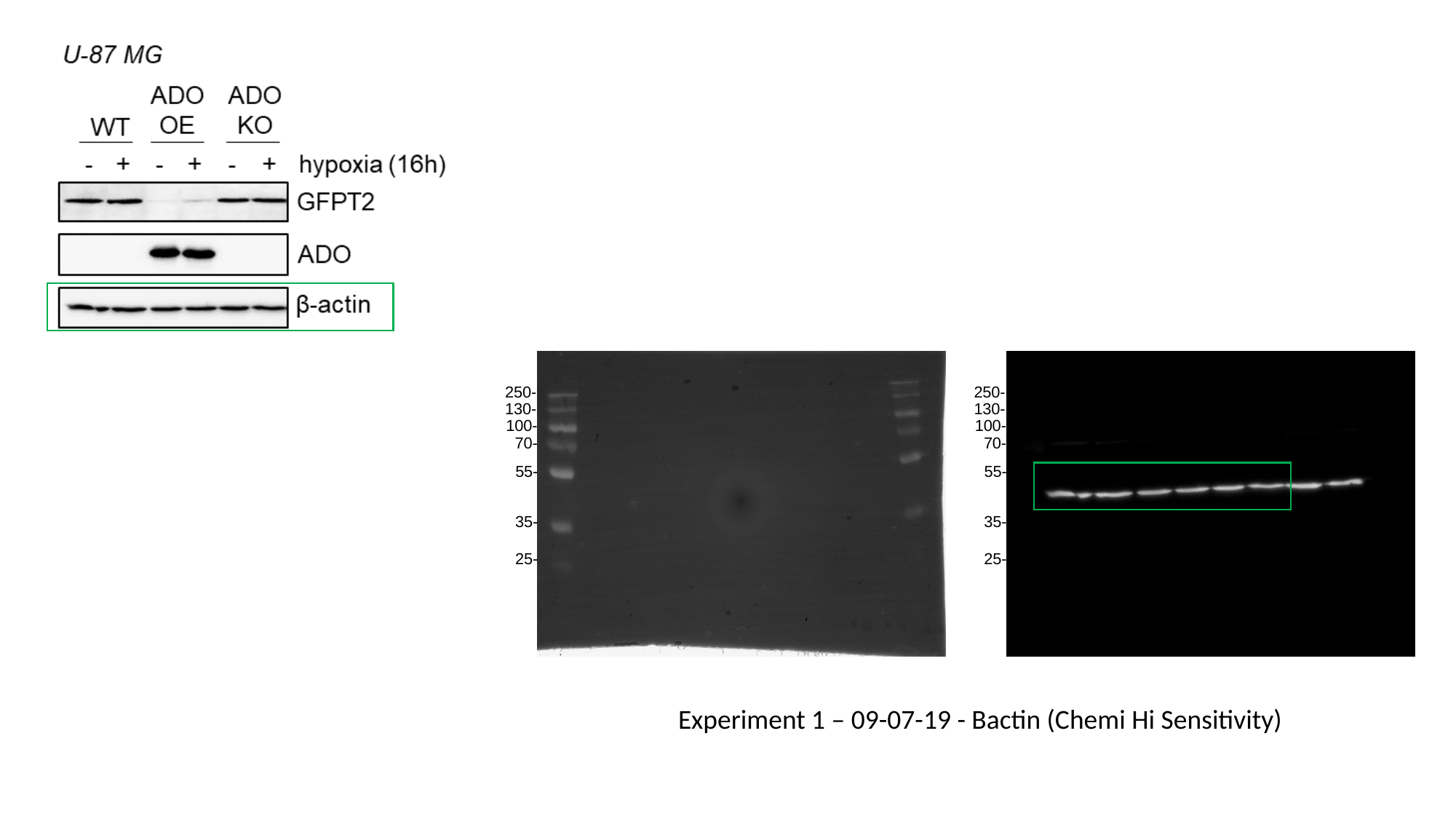

250-
250-
130-
130-
100-
100-
70-
70-
55-
55-
35-
35-
25-
25-
Experiment 1 – 09-07-19 - Bactin (Chemi Hi Sensitivity)

## Slide 4
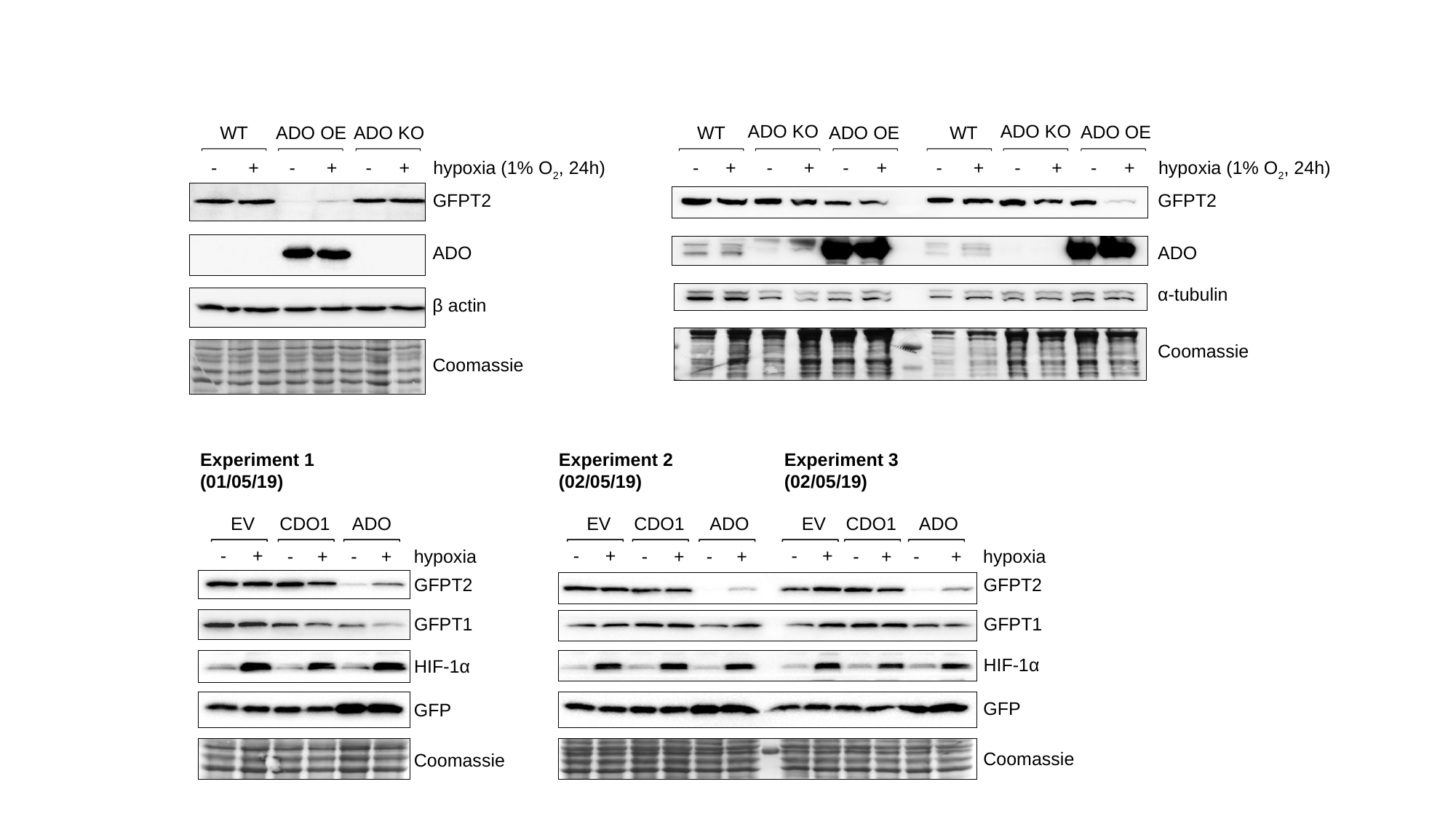

ADO KO
ADO KO
ADO OE
ADO OE
ADO KO
WT
ADO OE
WT
WT
hypoxia (1% O2, 24h)
hypoxia (1% O2, 24h)
-
+
-
+
-
+
-
+
-
+
-
+
-
+
-
+
-
+
GFPT2
GFPT2
ADO
ADO
α-tubulin
β actin
Coomassie
Coomassie
Experiment 3 (02/05/19)
Experiment 1 (01/05/19)
Experiment 2 (02/05/19)
EV
CDO1
ADO
EV
CDO1
ADO
EV
CDO1
ADO
+
+
+
-
-
-
+
+
+
-
-
-
-
+
hypoxia
-
+
hypoxia
-
+
GFPT2
GFPT2
GFPT1
GFPT1
HIF-1α
HIF-1α
GFP
GFP
Coomassie
Coomassie
