# Supplementary material for: N-terminal cysteine acetylation and oxidation patterns may define protein stability
Source: Nat Commun. 2024 Jun 25;15:5360. doi: 10.1038/s41467-024-49489-2 (PMC11199558; doi:10.1038/s41467-024-49489-2)
Supplement: Supplementary file 10 — Source Data [file 41467_2024_49489_MOESM10_ESM.zip › NCOMMS-23-38359 Source Data/Figure 8C Source Data.pptx]

## Slide 1
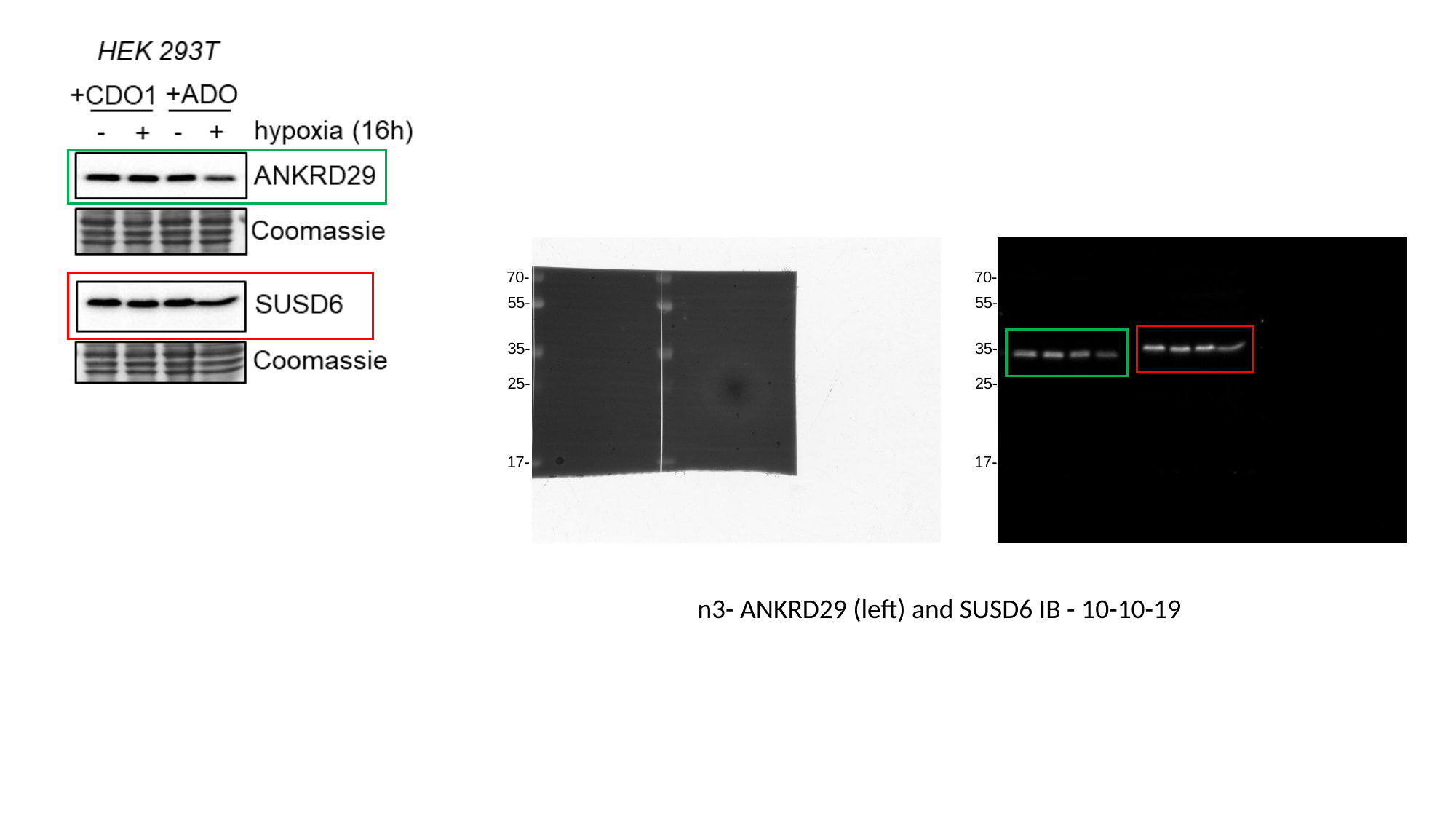

70-
70-
55-
55-
35-
35-
25-
25-
17-
17-
n3- ANKRD29 (left) and SUSD6 IB - 10-10-19

## Slide 2
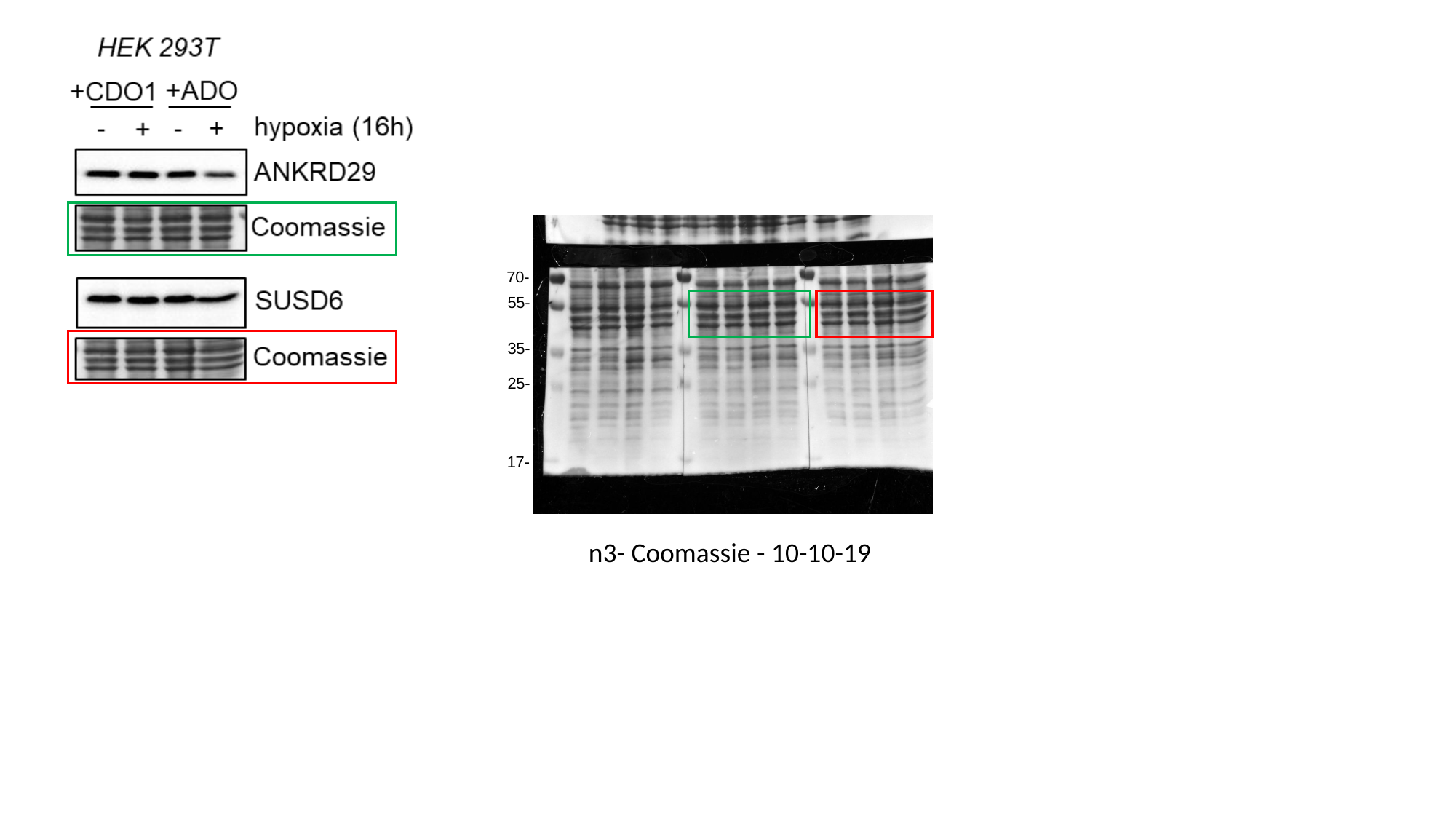

70-
55-
35-
25-
17-
n3- Coomassie - 10-10-19

## Slide 3
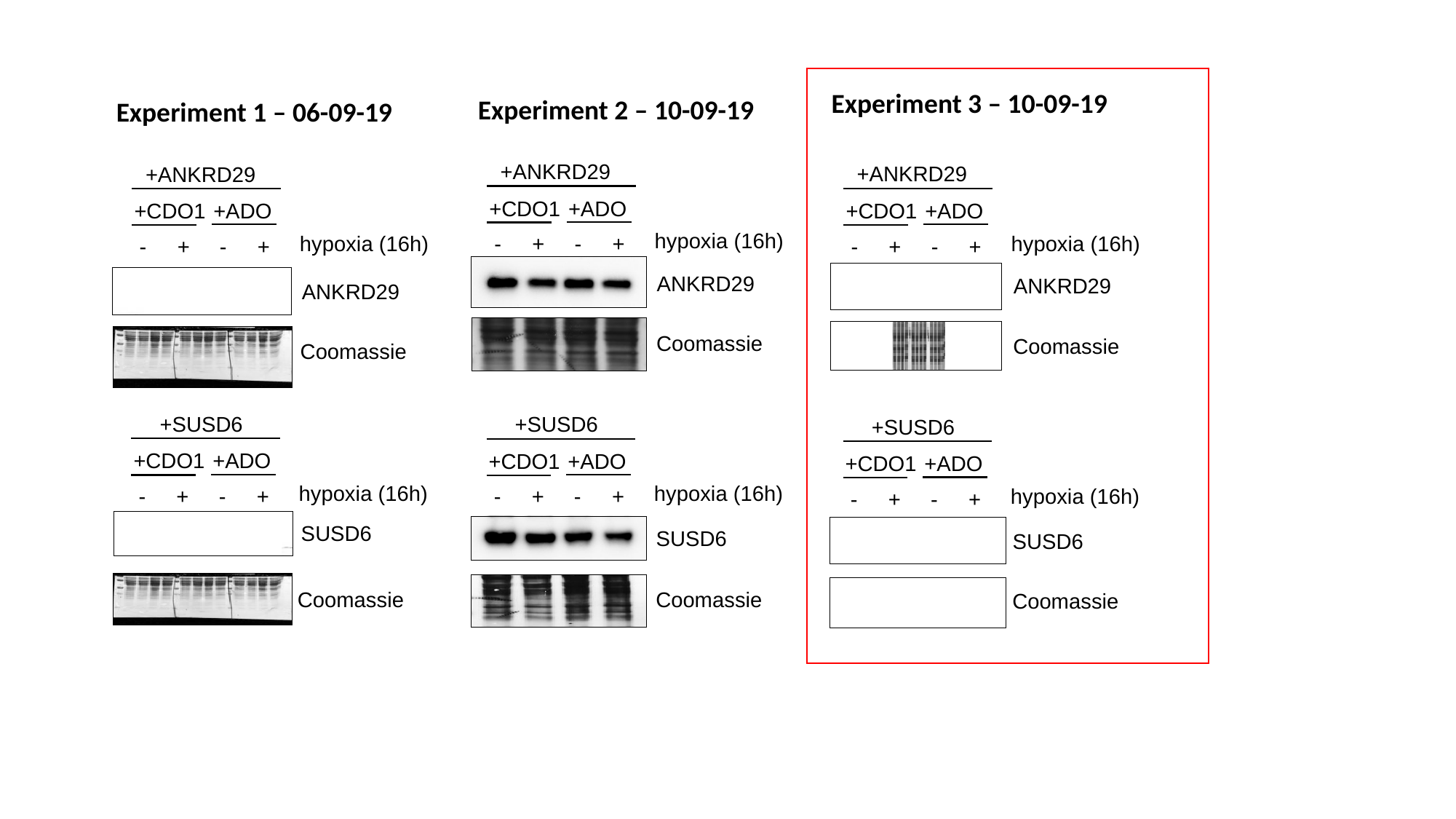

Experiment 3 – 10-09-19
Experiment 2 – 10-09-19
Experiment 1 – 06-09-19
+ANKRD29
+ANKRD29
+ANKRD29
+CDO1
+ADO
+CDO1
+ADO
+CDO1
+ADO
hypoxia (16h)
hypoxia (16h)
hypoxia (16h)
-
+
-
+
-
+
-
+
-
+
-
+
ANKRD29
ANKRD29
ANKRD29
Coomassie
Coomassie
Coomassie
+SUSD6
+SUSD6
+SUSD6
+CDO1
+ADO
+CDO1
+ADO
+CDO1
+ADO
hypoxia (16h)
hypoxia (16h)
hypoxia (16h)
-
+
-
+
-
+
-
+
-
+
-
+
SUSD6
SUSD6
SUSD6
Coomassie
Coomassie
Coomassie
