# Supplementary material for: N-terminal cysteine acetylation and oxidation patterns may define protein stability
Source: Nat Commun. 2024 Jun 25;15:5360. doi: 10.1038/s41467-024-49489-2 (PMC11199558; doi:10.1038/s41467-024-49489-2)
Supplement: Supplementary file 10 — Source Data [file 41467_2024_49489_MOESM10_ESM.zip › NCOMMS-23-38359 Source Data/Figure 8E Source Data.pptx]

## Slide 1
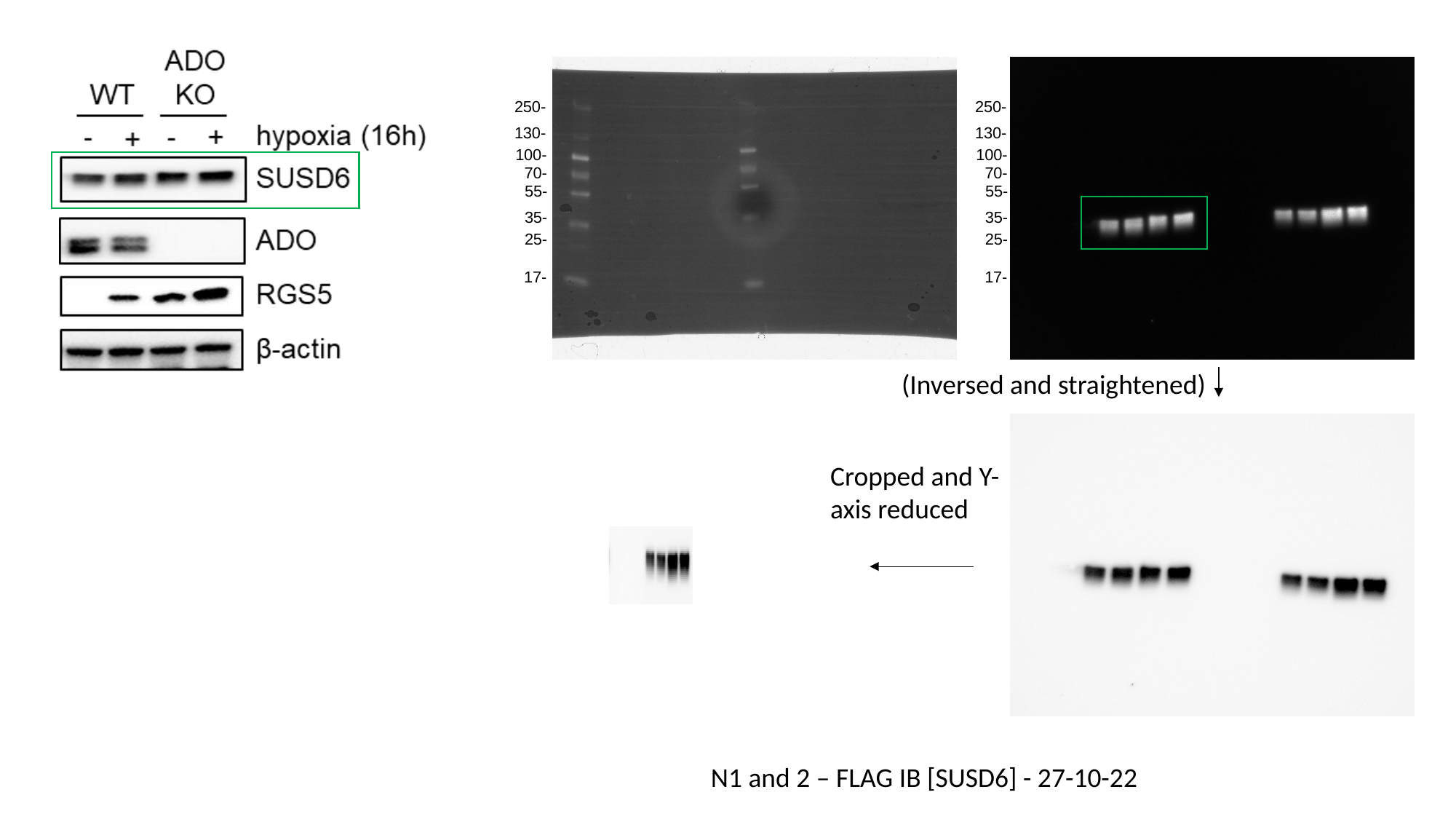

250-
250-
130-
130-
100-
100-
70-
70-
55-
55-
35-
35-
25-
25-
17-
17-
(Inversed and straightened)
Cropped and Y-axis reduced
N1 and 2 – FLAG IB [SUSD6] - 27-10-22

## Slide 2
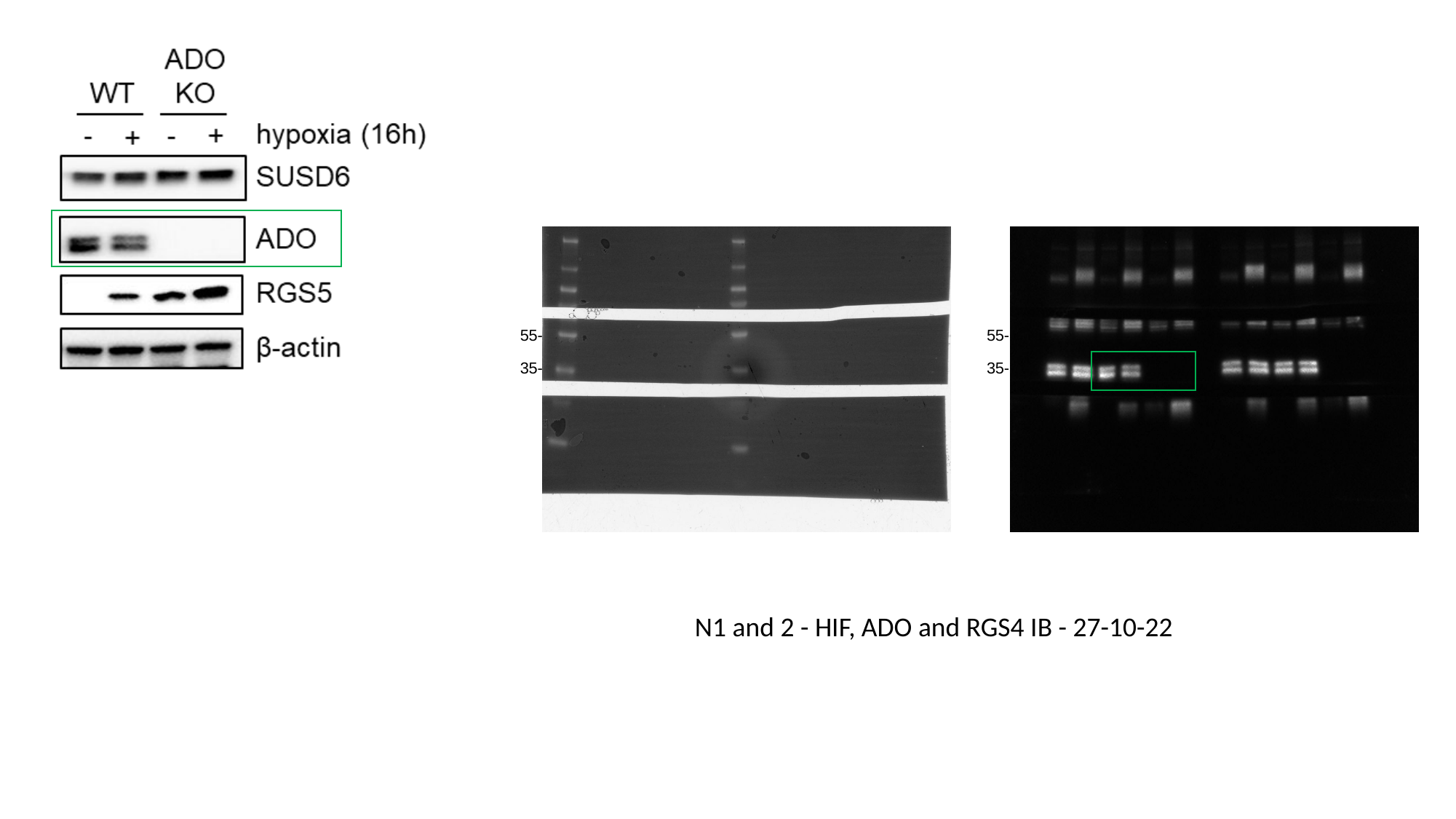

55-
55-
35-
35-
N1 and 2 - HIF, ADO and RGS4 IB - 27-10-22

## Slide 3
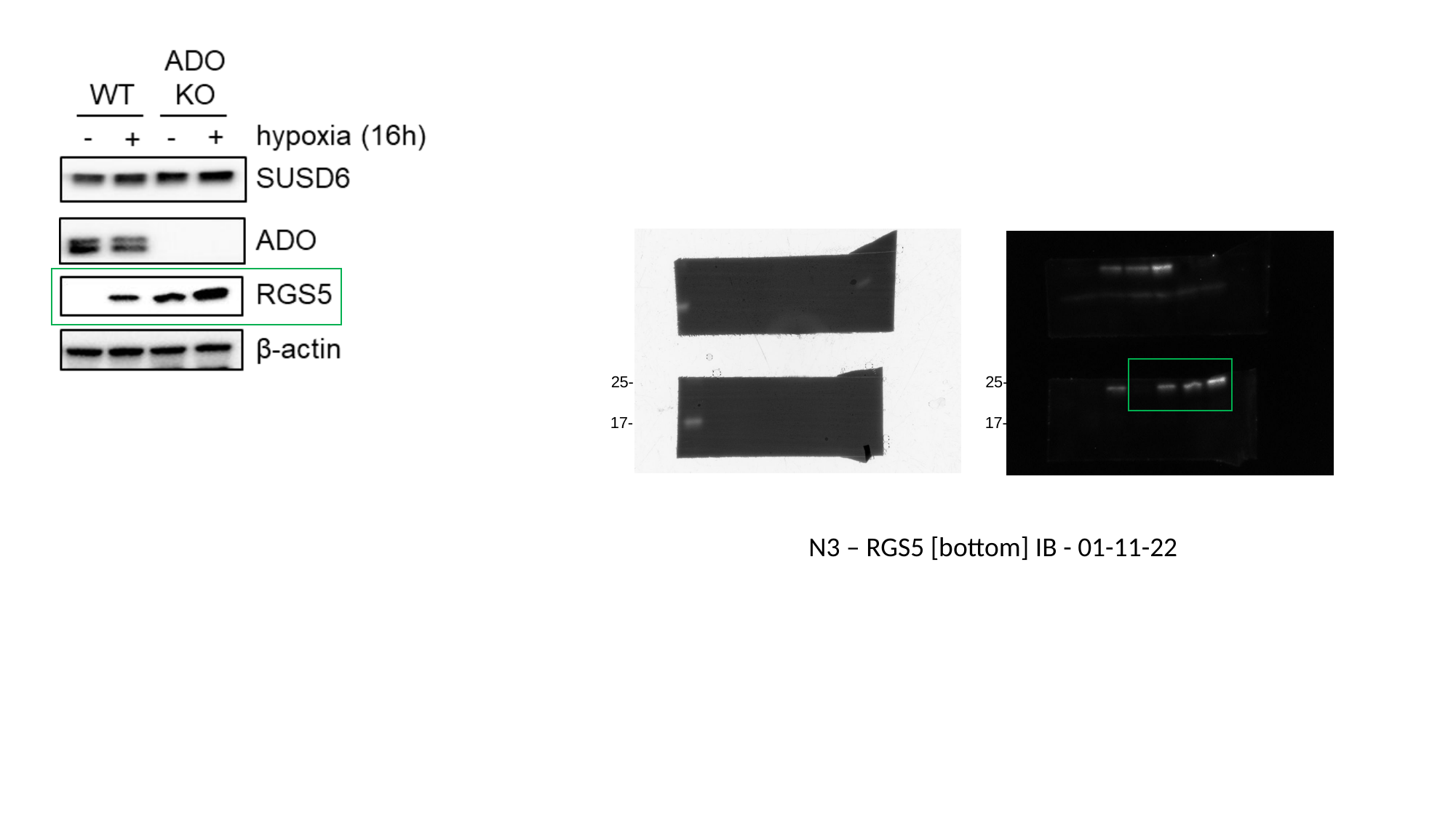

25-
25-
17-
17-
N3 – RGS5 [bottom] IB - 01-11-22

## Slide 4
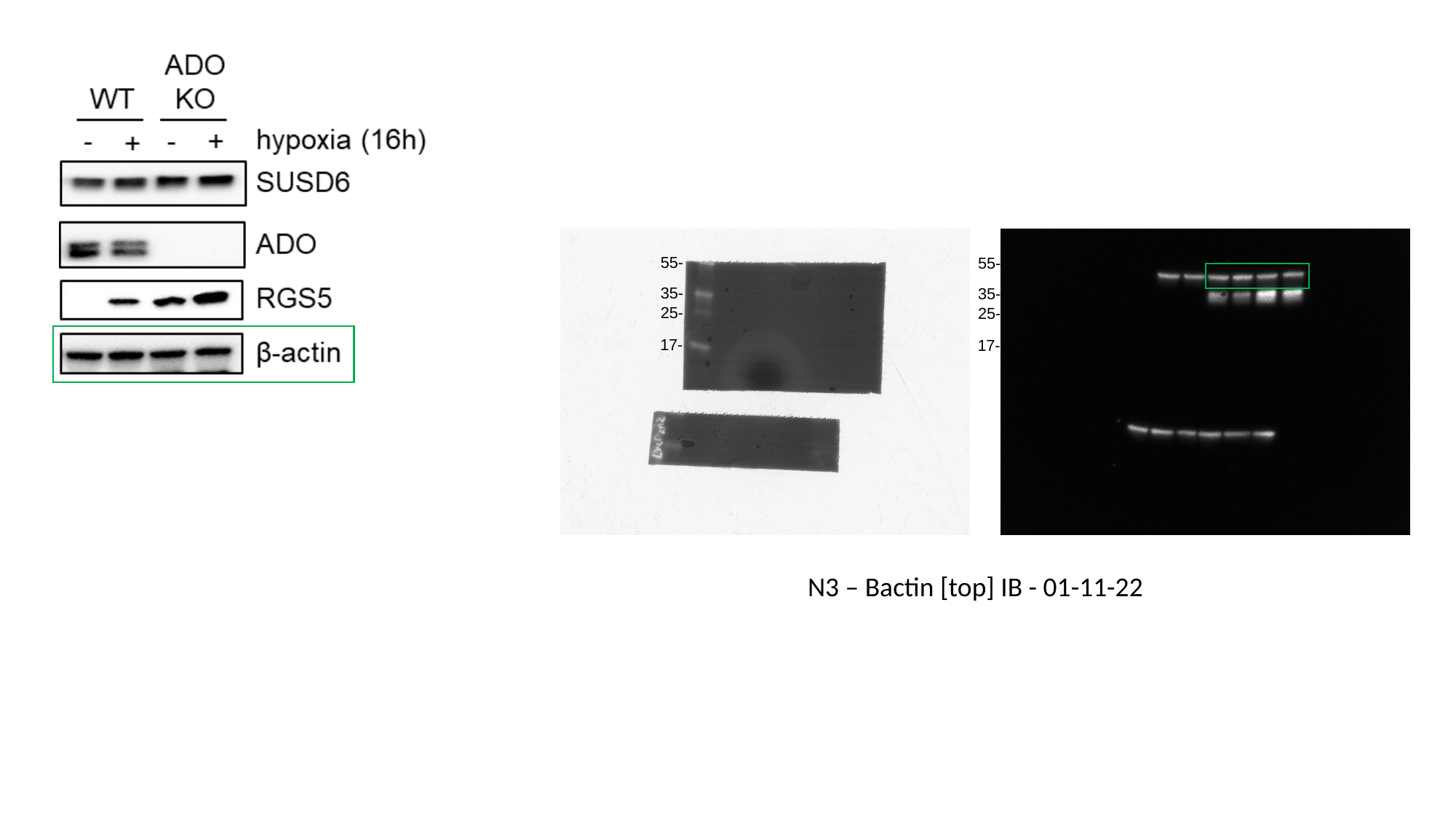

55-
55-
35-
35-
25-
25-
17-
17-
N3 – Bactin [top] IB - 01-11-22

## Slide 5
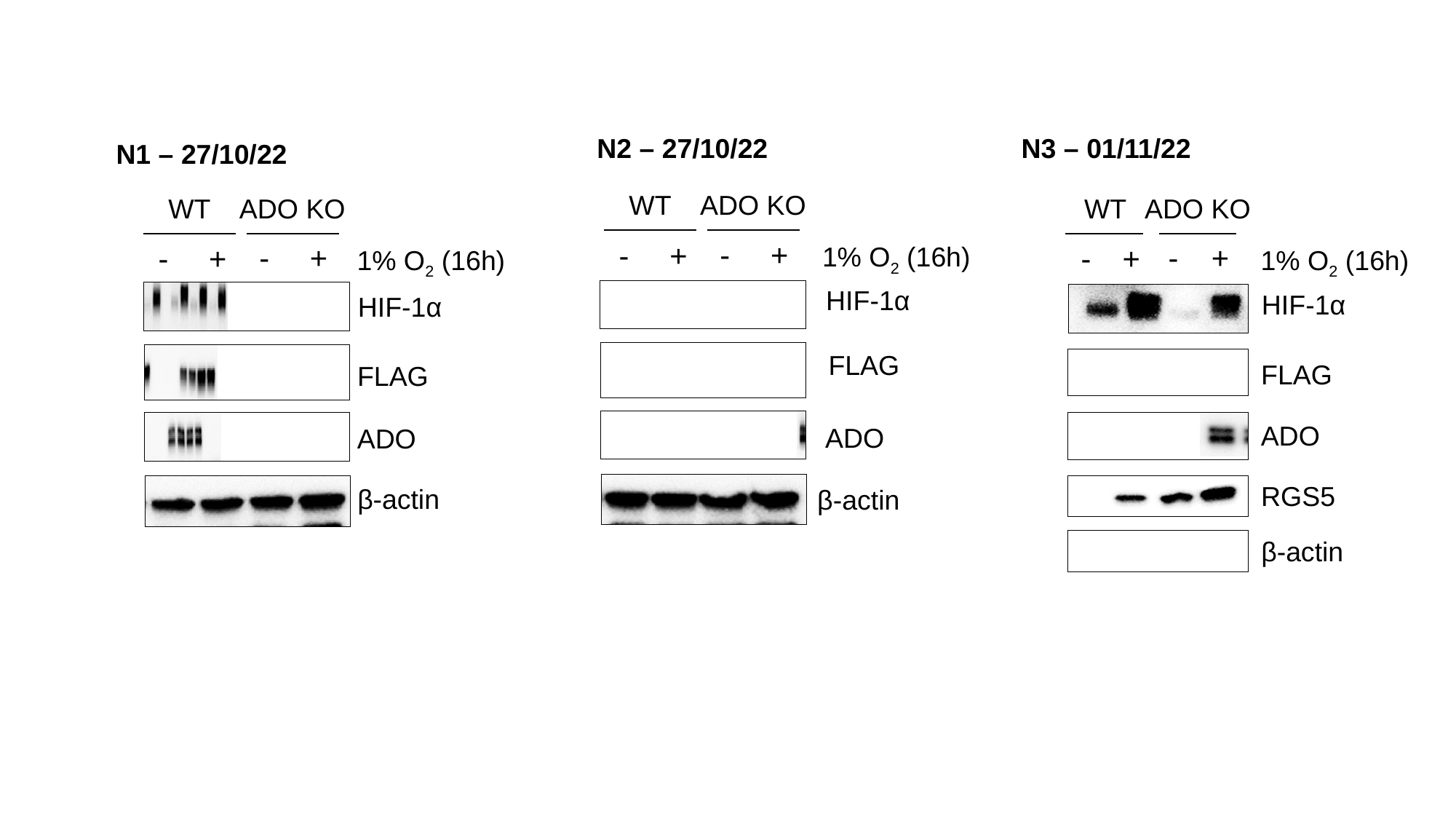

N2 – 27/10/22
N3 – 01/11/22
N1 – 27/10/22
ADO KO
WT
ADO KO
ADO KO
WT
WT
-
+
-
+
-
+
-
+
-
+
-
+
1% O2 (16h)
1% O2 (16h)
1% O2 (16h)
HIF-1α
HIF-1α
HIF-1α
FLAG
FLAG
FLAG
ADO
ADO
ADO
RGS5
β-actin
β-actin
β-actin
