# Supplementary material for: N-terminal cysteine acetylation and oxidation patterns may define protein stability
Source: Nat Commun. 2024 Jun 25;15:5360. doi: 10.1038/s41467-024-49489-2 (PMC11199558; doi:10.1038/s41467-024-49489-2)
Supplement: Supplementary file 10 — Source Data [file 41467_2024_49489_MOESM10_ESM.zip › NCOMMS-23-38359 Source Data/Supplementary Figure 8A Source dAta.pptx]

## Slide 1
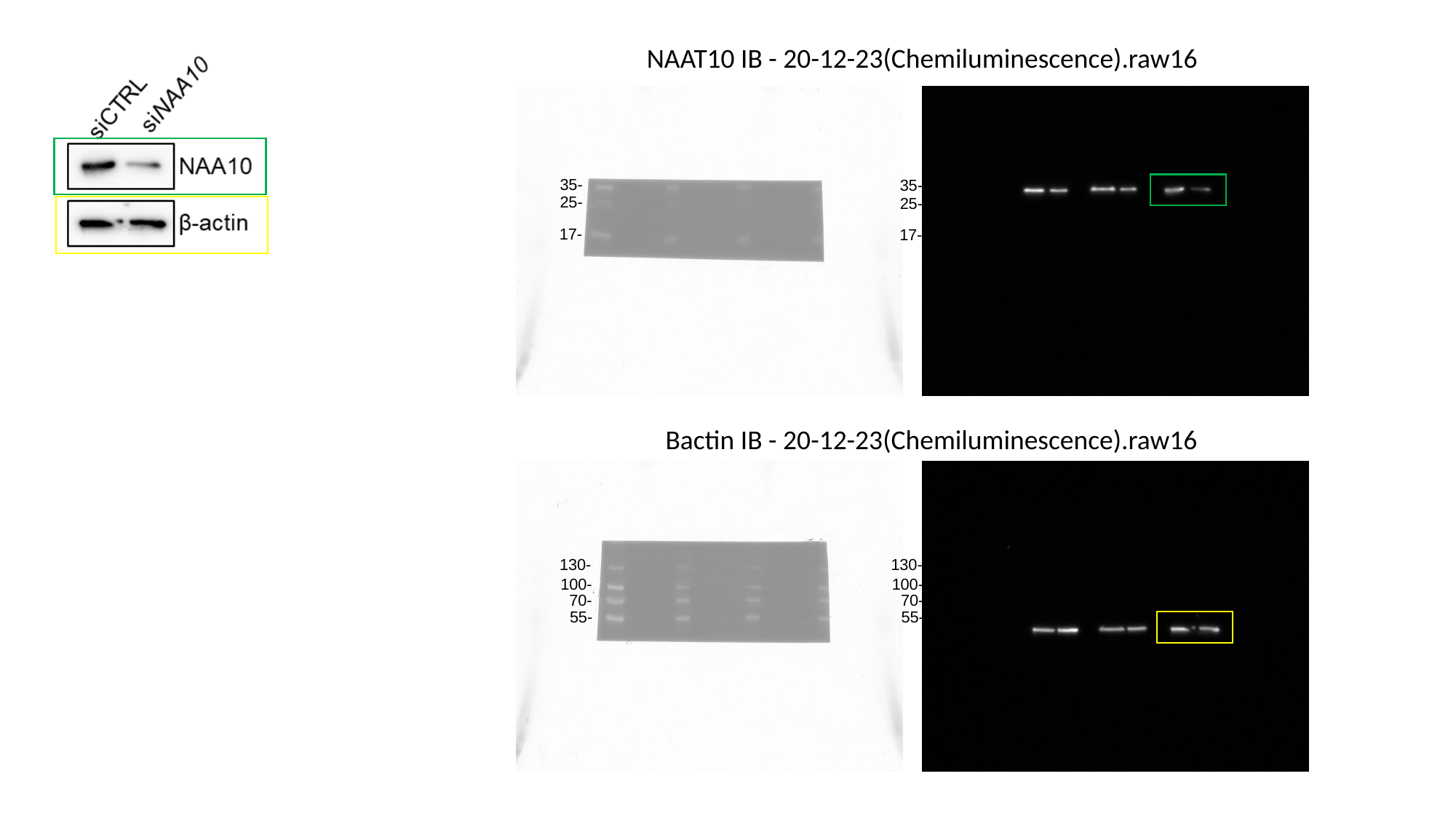

NAAT10 IB - 20-12-23(Chemiluminescence).raw16
35-
35-
25-
25-
17-
17-
Bactin IB - 20-12-23(Chemiluminescence).raw16
130-
130-
100-
100-
70-
70-
55-
55-

## Slide 2
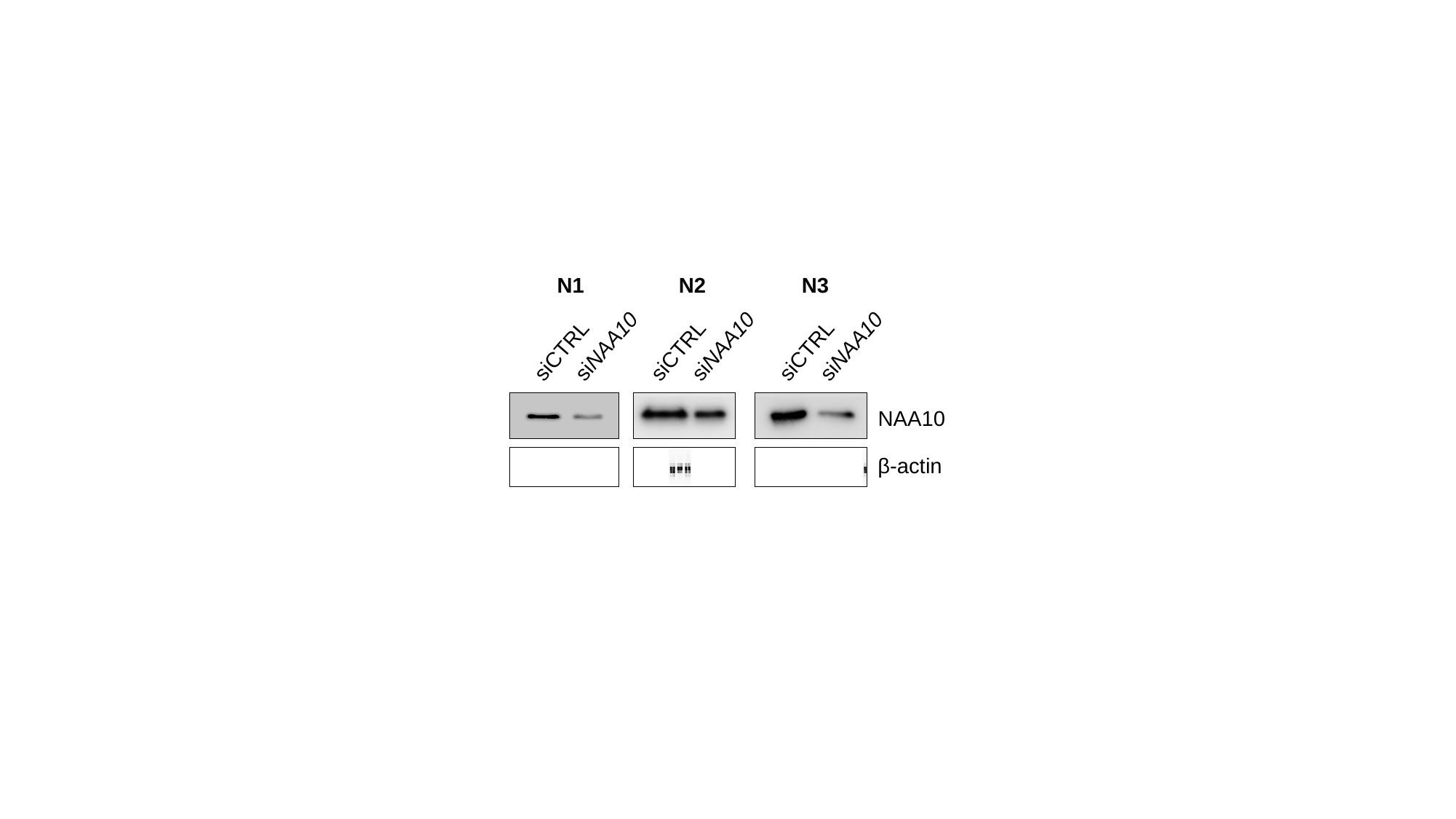

N1
N2
N3
siNAA10
siNAA10
siNAA10
siCTRL
siCTRL
siCTRL
NAA10
β-actin
